# Comparative profiling of cortical gene expression in Alzheimer’s disease patients and mouse models demonstrates a link between amyloidosis and neuroinflammation

**DOI:** 10.1038/s41598-017-17999-3

**Published:** 2017-12-19

**Authors:** Erika Castillo, Julio Leon, Guianfranco Mazzei, Nona Abolhassani, Naoki Haruyama, Takashi Saito, Takaomi Saido, Masaaki Hokama, Toru Iwaki, Tomoyuki Ohara, Toshiharu Ninomiya, Yutaka Kiyohara, Kunihiko Sakumi, Frank M. LaFerla, Yusaku Nakabeppu

**Affiliations:** 1 Division of Neurofunctional Genomics, Department of Immunobiology and Neuroscience, Medical Institute of Bioregulation, Kyushu University, 3-1-1 Maidashi, Higashi-ku, Fukuoka, 812–8582 Japan; 2 Laboratory for Proteolytic Neuroscience, RIKEN Brain Science Institute, Saitama, Japan; 3 Department of Neuropathology, Neurological Institute, Graduate School of Medical Sciences, Kyushu University, 3-1-1 Maidashi, Higashi-ku, Fukuoka, 812–8582 Japan; 4 Department of Neuropsychiatry, Graduate School of Medical Sciences, Kyushu University, 3-1-1 Maidashi, Higashi-ku, Fukuoka, 812–8582 Japan; 5 Department of Epidemiology and Public Health, Graduate School of Medical Sciences, Kyushu University, 3-1-1 Maidashi, Higashi-ku, Fukuoka, 812–8582 Japan; 6 Hisayama Research Institute for Lifestyle Diseases, Hisayama, Fukuoka, Japan; 7 Department of Neurobiology and Behavior, University of California, Irvine, CA 92697 USA; 8 Present Address: Department of Neurology, University of California, San Francisco, San Francisco, CA 94158 USA; 9 Present Address: Department of Neurosurgery, Japan Community Health Care Organization Kyushu Hospital, Kitakyushu, 806–8501 Japan

**Keywords:** Alzheimer's disease, Alzheimer's disease

## Abstract

Alzheimer’s disease (AD) is the most common form of dementia, characterized by accumulation of amyloid β (Aβ) and neurofibrillary tangles. Oxidative stress and inflammation are considered to play an important role in the development and progression of AD. However, the extent to which these events contribute to the Aβ pathologies remains unclear. We performed inter-species comparative gene expression profiling between AD patient brains and the *App* ^NL-G-F/NL-G-F^ and 3xTg-AD-H mouse models. Genes commonly altered in *App* ^NL-G-F/NL-G-F^ and human AD cortices correlated with the inflammatory response or immunological disease. Among them, expression of AD-related genes (*C4a*/*C4b*, *Cd74*, *Ctss*, *Gfap*, *Nfe2l2*, *Phyhd1*, *S100b*, *Tf*, *Tgfbr2*, and *Vim*) was increased in the *App* ^NL-G-F/NL-G-F^ cortex as Aβ amyloidosis progressed with exacerbated gliosis, while genes commonly altered in the 3xTg-AD-H and human AD cortices correlated with neurological disease. The *App* ^NL-G-F/NL-G-F^ cortex also had altered expression of genes (*Abi3*, *Apoe*, *Bin2*, *Cd33*, *Ctsc*, *Dock2*, *Fcer1g*, *Frmd6*, *Hck*, *Inpp5D*, *Ly86*, *Plcg2*, *Trem2*, *Tyrobp*) defined as risk factors for AD by genome-wide association study or identified as genetic nodes in late-onset AD. These results suggest a strong correlation between cortical Aβ amyloidosis and the neuroinflammatory response and provide a better understanding of the involvement of gender effects in the development of AD.

## Introduction

Dementia affects over 47 million people throughout the world, and this number is likely to increase to more than 131 million by 2050^1^. Alzheimer’s disease (AD) is the most common form of dementia, and amyloid β (Aβ) plaques and neurofibrillary tangles (NFTs) are the classical hallmarks of this disease^2^. Currently, a growing body of evidence supports the concept that oxidative stress and inflammation may also play an important role in the development and progression of AD pathologies. Data from clinical studies revealed systemic immune-related changes in AD brains^3–6^. However, whether those events occur during the later stages of disease or contribute to the Aβ pathologies remains unclear.

To better understand the molecular mechanisms of AD pathologies, different animal models have been established. Transgenic mouse models overexpress genetically modified Aβ precursor protein (APP), presenilin (PSEN) and/or the microtubule-associated protein tau (MAPT), to induce accumulation of Aβ or neuronal dysfunction. However, these transgenic mouse models develop AD-like pathologies at different ages and to different extents due to expression levels of AD-related proteins that are dependent on promoters used in transgene constructs, as well as copy number of transgenes and inserted regions^7–9^. To more accurately reproduce AD pathologies, *App* knock-in mouse models that carry pathogenic mutation(s) in *App* and/or *Psen1* genes have been established. These mouse models show age-dependent amyloidosis, with activated astrocytes and microglia surrounding Aβ plaques, synaptic dysfunction and deficits in behavioural and cognition assays; revealing that amyloidosis triggered by pathological modifications in APP processing is sufficient to induce cognitive impairment^8,10,11^.

Most studies in mouse models have focused on the effect of AD pathologies in the hippocampal area. However, cortical areas also play an important role in the maintenance of brain integrity; novel imaging technologies show Aβ depositions and morphological alterations in the cortex of AD patients^12–16^, raising the question of how Aβ accumulation in the brain cortex is involved in pathophysiological alterations observed in AD.

The present study aimed to identify expression profiles of cortical genes in AD patients and AD mouse models, as well as their associated biological functions. We performed inter-species comparative gene expression profiling between AD patient brains and the *App* ^NL-G-F/NL-G-F^ and 3xTg-AD (3xTg-AD-H) mouse models to determine differential gene expression profiles to understand how expression changes contribute to the progression of AD pathologies. *App* ^NL-G-F/NL-G-F^ mice carrying the homozygous mutant *App* gene encoding the humanised Aβ sequence (G601R, F606Y, and R609H) with three pathogenic mutations, namely Swedish (KM595/596NL), Beyreuther/Iberian (I641F), and Arctic (E618G)^10^, progressively exhibit Aβ accumulation starting at 4 to 6 months of age, dense distributions of microglia and astrocytes from 9 months of age, and behavioural symptoms from 8 to 12 months of age^10,11^. The 3xTg-AD-H mice that overexpress two mutated human transgenes, Swedish *APP* (KM670/671NL) and *MAPT* (P301L) driven by the exogenous neuronal *Thy*1.2 promoter, with a knock-in mutation of *Psen1* (M146V) that promotes formation of Aβ plaques and NFTs, also exhibit behavioural symptoms and Aβ and Tau pathologies before 12 months of age^17^. It is noteworthy that the Tau pathology that occurs in the 3xTg-AD-H brain is induced by a pathogenic Tau protein encoded by a mutant *MAPT* (P301L) gene, not as a result of elevated Aβ, therefore this AD mouse model enables us to examine the brain response to Tau pathology.

We thus examined the two mouse models, and the gene expression profiles altered by only Aβ, or by Aβ and Tau pathologies, were compared with those obtained from human AD brains. We found that *App* ^NL-G-F/NL-G-F^ mice, but not 3xTg-AD-H mice, exhibited an altered expression profile of cortical genes, indicating a strong correlation between cortical Aβ amyloidosis and the neuroinflammatory response, similar to that observed in the human AD cortex.

## Results

### Altered gene expression profiles in cortices of AD patients and AD mouse models

Previously, we obtained gene expression profiles from three human brain regions––hippocampus and the temporal and frontal cortices––prepared from post-mortem brains of AD subjects, and found a significant alteration in the hippocampal gene expression profile with AD pathologies^18^. In the present study, we aimed to characterise gene expression profiles in AD cortical regions by re-analysing the microarray data from temporal and frontal cortices of AD patients and controls (Supplementary Tables S1 and S2), using the Affymetrix Expression Console and Transcriptome Analysis Console (TAC) software.

**Figure 1:**
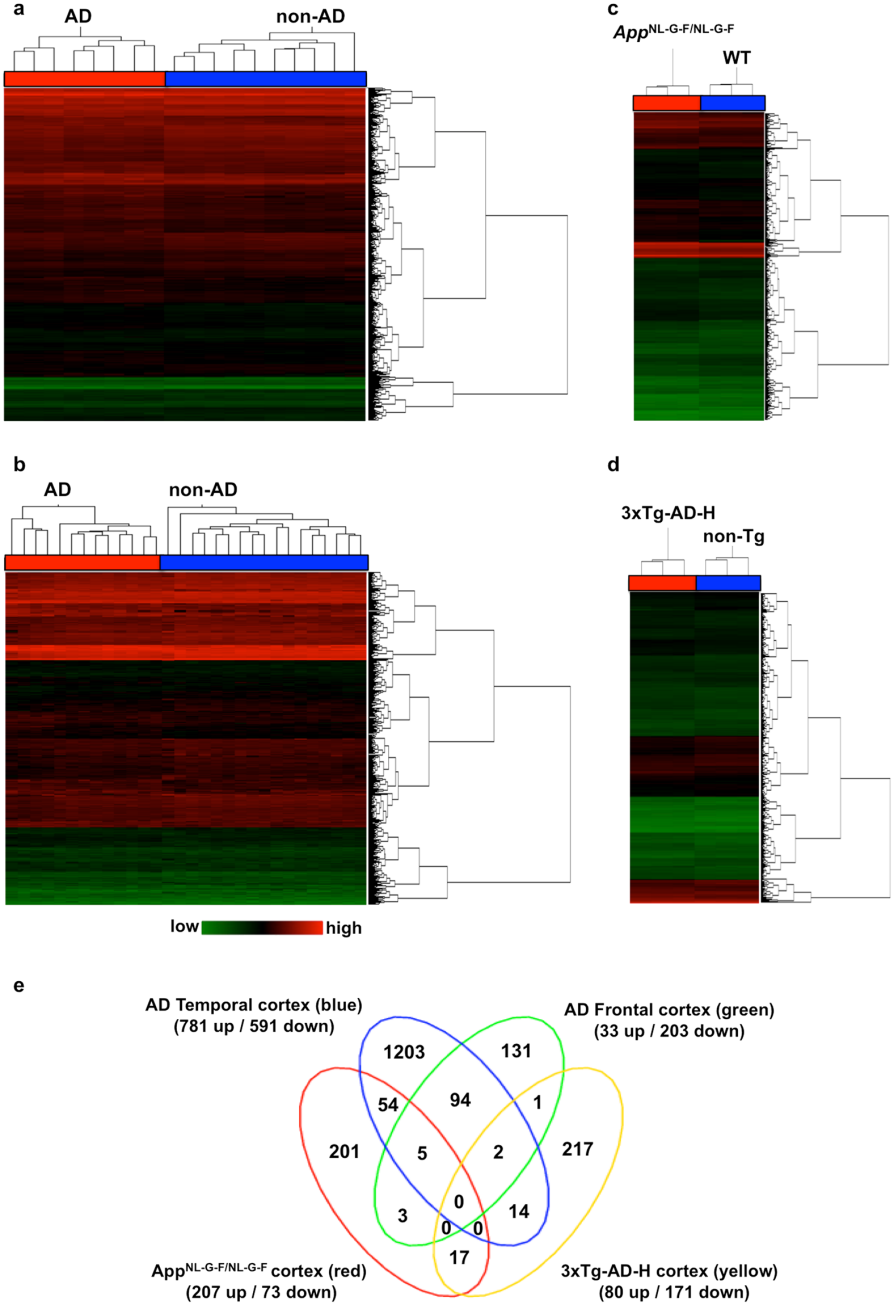
Altered gene expression profiles in cortices of AD patients and AD mouse models. Lists of transcript clusters that exhibit significant alterations between AD patients and non-AD subjects or between AD mouse model and its control, obtained from microarray data (ANOVA: *P* < 0.05, fold change ≥ 1.2 or ≤−1.2), were subjected to hierarchical clustering analysis: (**a**) human AD temporal cortices, (**b**) human AD frontal cortices, (**c**) *App* ^NL-G-F/NL-G-F^ mouse cortices, (**d**) 3xTg-AD-H mouse cortices, compared with each control. Red columns indicate data from AD patients, *App* ^NL-G-F/NL-G-F^ and 3xTg-AD-H mice, blue columns represent data from each control. Levels of gene expression are shown in green (low) to red (high). (**e**) Venn diagram shows overlapping genes with significantly altered expression in each comparison (ANOVA: *P* < 0.05, log_2_ > 6.64, fold change ≥ 1.2 or ≤−1.2), between and among the four sets of comparisons. Total number of up- and downregulated genes in each group is shown in parentheses.

As shown in Fig. 1a and b, the temporal (8 AD and 10 non-AD cases) and frontal (13 AD and 17 non-AD cases) samples with no overlapped distribution in the Principal Component Analysis (PCA) exhibited clear separation of AD and non-AD cases by hierarchical clustering of their expression profiles (Supplementary Figs S1 and S2). By analysing expression profiles of these subjects using TAC and Ingenuity Pathway Analysis (IPA) software, we found that 1372 (781 up, 591 down) genes in the temporal cortex and 236 (33 up, 203 down) genes in the frontal cortex were differentially expressed between AD and non-AD cases (ANOVA: *P* < 0.05, a lower bi-weight average signal (log_2_) > 6.64, a fold change ≥ 1.2 or ≤−1.2) (Supplementary Tables S3 and S4). We then validated the microarray data of 10 transcripts by real-time quantitative RT-PCR (qRT-PCR) analyses (primers shown in Supplementary Table S5) in six AD (3 males and 3 females) and six non-AD (3 males and 3 females) samples from both temporal and frontal cortices. This showed that the obtained averaged fold-change values in genes between AD and non-AD samples highly correlated with corresponding data obtained from microarray analyses (Supplementary Fig. S3).

Next, we performed microarray analyses using cortical RNA prepared from 12-month-old *App* ^NL-G-F/NL-G-F^ and 3xTg-AD-H mice, together with their respective control mice. Both models exhibited a clear separation from their controls by hierarchical clustering of their expression profiles (Fig. 1c,d). By analysing the expression profiles of the two AD mouse models with TAC and IPA, we found that 280 (207 up, 73 down) genes in the *App* ^NL-G-F/NL-G-F^ mice and 251 (80 up, 171 down) genes in the 3xTg-AD-H mice were differentially expressed compared with the corresponding controls (ANOVA: *P* < 0.05, a lower bi-weight average signal (log_2_) > 6.64, a fold change ≥ 1.2 or ≤−1.2) (Supplementary Tables S6 and S7). We again validated these microarray data by qRT-PCR analyses of the 10 transcripts in all samples, showing good correlations of values of fold change (AD model vs. control) between the two measurements (Supplementary Fig. S3).

We then compared the gene lists among all four groups, and found that only 17 genes were commonly altered between *App* ^NL-G-F/NL-G-F^ and 3xTg-AD-H cortices, and none of the genes were significantly altered in human AD cortices. A total of 62 genes were shared between the *App* ^NL-G-F/NL-G-F^ cortex and human cortices, 54 with only temporal, 3 with only frontal, and 5 with both cortical areas (Fig. 1e, Supplementary Table S8). However, the 3xTg-AD-H cortex shared a total of 17 genes with human cortices, 14 with only temporal, 1 with only frontal, and 2 with both (Fig. 1e, Supplementary Table S9). These data suggest that *App* ^NL-G-F/NL-G-F^ and 3xTg-AD-H cortices represented different aspects of AD pathologies, and that the *App* ^NL-G-F/NL-G-F^ cortex more closely represented the gene expression profile observed in the temporal cortex of AD patients.

### The *App*^NL-G-F/NL-G-F^ cortex, and to a lesser extent the temporal cortex of AD patients, exhibit increased expression of genes related to glial activation

We compared expression levels of genes encoding specific markers for four major types of brain cells: astrocytes, microglia, neurons and oligodendrocytes (Table 1), in order to evaluate changes in cell populations in AD brains. Relative expression levels of some markers related to activation states of astrocytes (*Aqp4*, *Gfap*) and microglia (*Cd68*, *Itgam*) were significantly increased in the human AD temporal cortex and more prominently in the *App* ^NL-G-F/NL-G-F^ cortex, suggesting gliosis. These trends were barely observed in human AD frontal and 3xTg-AD-H cortices. Expression levels of some oligodendrocyte markers were also significantly increased in human AD temporal cortex, and to a lesser extent in *App* ^NL-G-F/NL-G-F^ cortex. Most neuronal markers exhibited a trend towards decreased expression in human AD cortices. In particular, the expression levels of *RBFOX3* encoding neuronal nuclear antigen (NeuN), a marker for post-mitotic neurons, were 18 to 27% lower than non-AD controls, thus supporting the neuronal loss observed in AD cortices. In contrast, there was no significant reduction in the expression of any neuronal marker in the two AD mouse models, in good agreement with the observation that these AD mouse models do not exhibit neuronal loss in the brains^7,10,17^, indicating that the stages of disease being compared between human and mouse brains is not the same.

**Table 1:** Altered expression of gene markers for various brain cell types in cortices of AD patients and AD mouse models.

Cell Type	Gene Symbol	Relative expression (% to control)			
		AD temporal	AD frontal	*App*^NL-G-F/NL-G-F^	3xTg-AD-H
Astrocytes	*ALDH1L1*	**148**.**45**	113.29	104.97	95.26
	*AQP4*	**122**.**26**	104.25	**128**.**34**	107.92
	*GFAP*	**164**.**72**	**123**.**11**	**373**.**21**	114.87
	*SLC1A2*	83.51	88.88	104.25	90.13
	*SLC1A3*	100.70	103.53	109.43	97.94
	*S100B*	**131**.**04**	100.00	**131**.**95**	90.75
	**Mean**	125.11	105.51	158.69	99.48
	**SD**	29.95	11.69	105.77	9.92
Microglia	*AIF1*	107.92	103.53	**136**.**60**	95.26
	*CD68*	**133**.**79**	113.29	**329**.**44**	102.81
	*CORO1A*	87.06	88.88	105.70	110.19
	*EMR1*	95.93	105.70	**122**.**26**	96.59
	*ITGAM*	111.73	107.92	**154**.**76**	**131**.**95**
	*LGALS3*	105.70	106.44	**131**.**95**	100.70
	**Mean**	107.02	104.29	163.45	106.25
	**SD**	15.89	8.23	82.91	13.66
Neurons	*CHGA*	95.93	92.02	94.61	98.62
	*ENO2*	88.88	97.27	107.18	97.27
	*NEFH*	97.27	95.26	101.40	92.66
	*NEFL*	**79**.**55**	93.30	105.70	93.95
	*NEFM*	82.93	90.75	**133**.**79**	84.67
	*RBFOX3*	**72**.**70**	81.79	104.97	97.94
	*SNAP25*	87.06	86.45	101.40	100.00
	*SYP*	85.86	88.88	104.97	102.81
	*SYT1*	87.66	90.75	90.75	99.31
	*TUBB1*	97.27	97.94	102.10	93.30
	*TUBB2A*	97.94	98.62	98.62	107.18
	*TUBB2B*	114.08	101.40	**121**.**42**	80.11
	*TUBB3*	94.61	106.44	103.53	97.94
	*TUBB4A*	94.61	94.61	93.95	105.70
	*TUBB4B*	98.62	90.13	100.00	105.70
	*TUBB6*	99.31	100.70	87.06	104.97
	**Mean**	92.14	94.14	103.22	97.63
	**SD**	9.68	6.20	11.28	7.50
Oligodendrocytes	*MAG*	**164**.**72**	100.00	**129**.**24**	101.40
	*MBP*	114.08	97.94	110.19	92.66
	*MOG*	**177**.**77**	100.70	105.70	117.28
	*SOX10*	111.73	97.27	94.61	92.66
	**Mean**	142.07	98.98	109.93	101.00
	**SD**	34.12	1.63	14.44	11.61

Significantly altered genes between AD vs. non-AD or AD mouse model vs control (ANOVA: *P* < 0.05, fold change ≥ 1.2 or ≤ −1.2) are indicated in bold.

Taken together, these data indicate aggressive gliosis in AD cortices, especially in the *App* ^NL-G-F/NL-G-F^ cortex, in accordance with previous reports^10,11^, suggesting that neuroinflammation in the *App* ^NL-G-F/NL-G-F^ cortex, with increased Aβ burden, may represent pathological alterations seen in the human AD temporal cortex.

### Functional analysis of commonly altered genes in cortices of AD patients and AD mouse models suggests significantly altered neuroinflammatory responses

**Figure 2:**
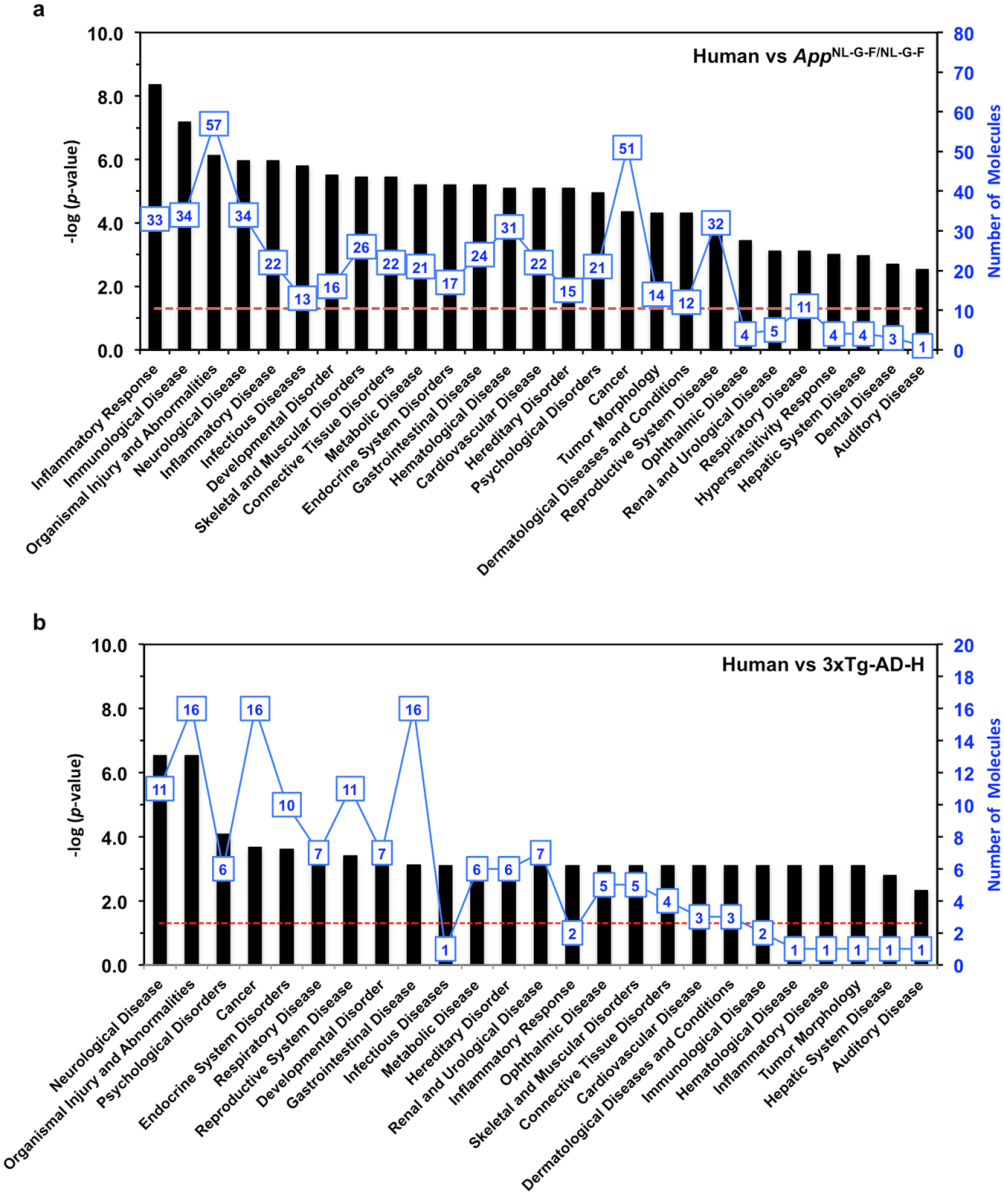
Biological functions of commonly altered genes in cortices of AD mouse models and AD patients. List of genes with commonly altered expression between the *App* ^NL-G-F/NL-G-F^ mouse and human AD cortices (frontal and temporal) shown in Supplementary Table S8 (**a**), and between the 3xTg-AD-H mouse and human AD cortices (frontal and temporal) shown in Supplementary Tables S9 (**b**), were subjected to IPA Core Analysis. In each graph, black bars indicate the *P*-value (−log [P-value]); blue lines indicate the number of molecules categorised in each biological function. The red dashed line indicates the threshold for *P*-value (−log [P-value] = 1.3).

A total of 62 genes commonly altered in the *App* ^NL-G-F/NL-G-F^ and human AD cortices were subjected to biological function analysis using IPA, and were categorised into various biofunctions: inflammatory response (33), immunological disease (34), organismal injury and abnormalities (57), neurological disease (34), inflammatory disease (22), and others (Fig. 2a, Supplementary Fig. S4). However, 17 genes with commonly altered expression in the 3xTg-AD-H mouse and human cortices were categorised into neurological disease (11), organismal injury and abnormalities (16), psychological disorders (6), cancer (16), endocrine system disorder (10), and others (Fig. 2b, Supplementary Fig. S5), suggesting that *App* ^NL-G-F/NL-G-F^ and 3xTg-AD-H cortices represented different aspects of the human AD pathologies.

**Figure 3:**
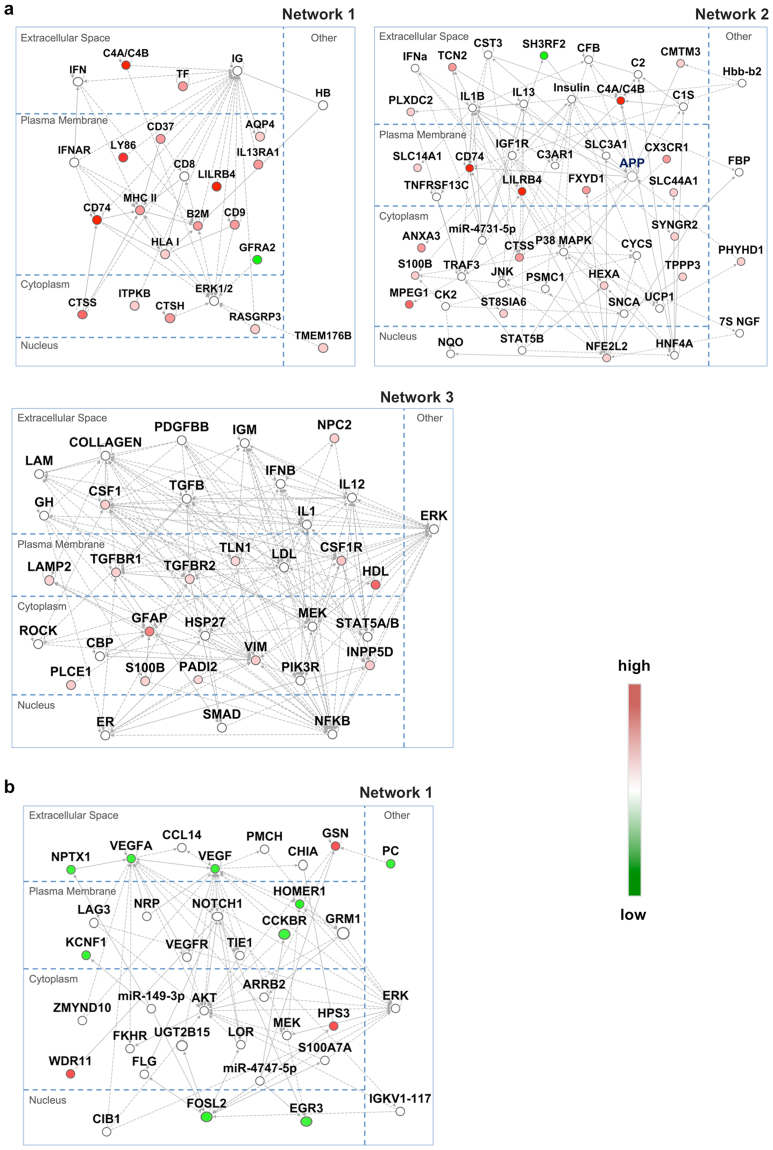
Top networks of commonly altered genes in cortices of two AD mouse models and AD patients. (**a**) The top 3 networks of genes with commonly altered expression between the *App* ^NL-G-F/NL-G-F^ mouse and human AD cortices (frontal and temporal) shown in Supplementary Table S8. Network 1 includes 17 upregulated genes and 1 downregulated gene. Network 2 includes 20 upregulated genes and 1 downregulated gene. Network 3 includes 14 upregulated genes in AD cortices. We included a dashed line to connect *Phyhd1* and *App* in Network 2, according to our results and a previous report^19^. **(b)** The top network of genes with commonly altered expression between the 3xTg-AD-H mouse and human AD cortices (frontal and temporal) shown in Supplementary Table S9. Network 1 includes 3 upregulated and 9 downregulated genes in AD cortices. Encoded molecules were placed in an appropriate subcellular compartment based on IPA, and “other” denotes unspecific or unknown localization. Solid lines indicate direct interactions and dashed lines indicate indirect interactions. Fold change is denoted as a green-white-red colour gradient, from green (downregulated) to red (upregulated).

We next applied the commonly altered genes between the *App* ^NL-G-F/NL-G-F^ and human AD cortices (Supplementary Table S8) into network prediction using IPA. Results showed that the most relevant network includes proteins encoded by 12 upregulated genes: *C4A/C4B*, *CD74*, *CTSS*, *TF*, the major histocompatibility complex (*MHC*), the human leukocyte antigen system (*HLA)*, *B2M*, *LILRB4*, *CD37*, *CD9*, *IL13RA1* and *AQP4* (Network 1, Fig. 3a), suggesting enhanced functions related to cell-cell signalling and humoral immune response. The second-most relevant network includes 13 upregulated molecules related to the inflammatory response (*ANXA3*, *C4A/C4B*, *CD74*, *CTSS*, *CX3CR1*, *HEXA*, *LILRB4*, *MPEG1*, *NFE2L2*, *PHYHD1*, *S100B*, *ST8SIA6* and *SYNGR2*), which have direct or indirect connections with *APP* (Network 2, Fig. 3a). Among them, *PHYHD1* was previously identified as one of genes upregulated in association with Braak stages of human AD brains, and it is known to directly interact with Aβ42^19^. Increased expression of the *Phyhd1* gene in a mouse model was observed herein for the first time, in the *App* ^NL-G-F/NL-G-F^ mice, strongly suggesting functional involvement of PHYHD1 in AD pathology. The third network, related to organismal injury and cellular movement, contains 15 upregulated molecules, including GFAP, VIM, S100B, TGFBR2, TGFBR1, TLN1, LAMP2, CSF1 and CSF1R, involved in cytoskeletal arrangement, vacuolisation and activation of glial cells, as previously reported in human AD brains and mouse models^20–22^ (Network 3, Fig. 3a). Taken together, these data suggest that commonly altered genes between the *App* ^NL-G-F/NL-G-F^ and human AD cortices are functionally interconnected in molecular pathways that link AD pathologies, especially amyloidosis, to neuroinflammation.

Expression of 11 genes was commonly downregulated in the 3xTg-AD-H and human AD temporal cortices (Supplementary Table S9). Among them, *CCKBR*, *EGR3*, *FOSL2*, *HOMER1*, *KCNF1*, *NPTX1* and *VEGFA* constitute a network related to cardiovascular system development and function, organismal development, and cell signalling (Fig. 3b), and *CCKBR*, *EGR3*, *HOMER1* and *KCNF1* genes have been previously reported to be downregulated in human AD hippocampus^18^.

### Expression levels of common AD-related genes increased with amyloidosis progression in the cortex of *App*^NL-G-F/NL-G-F^ mice

**Figure 4:**
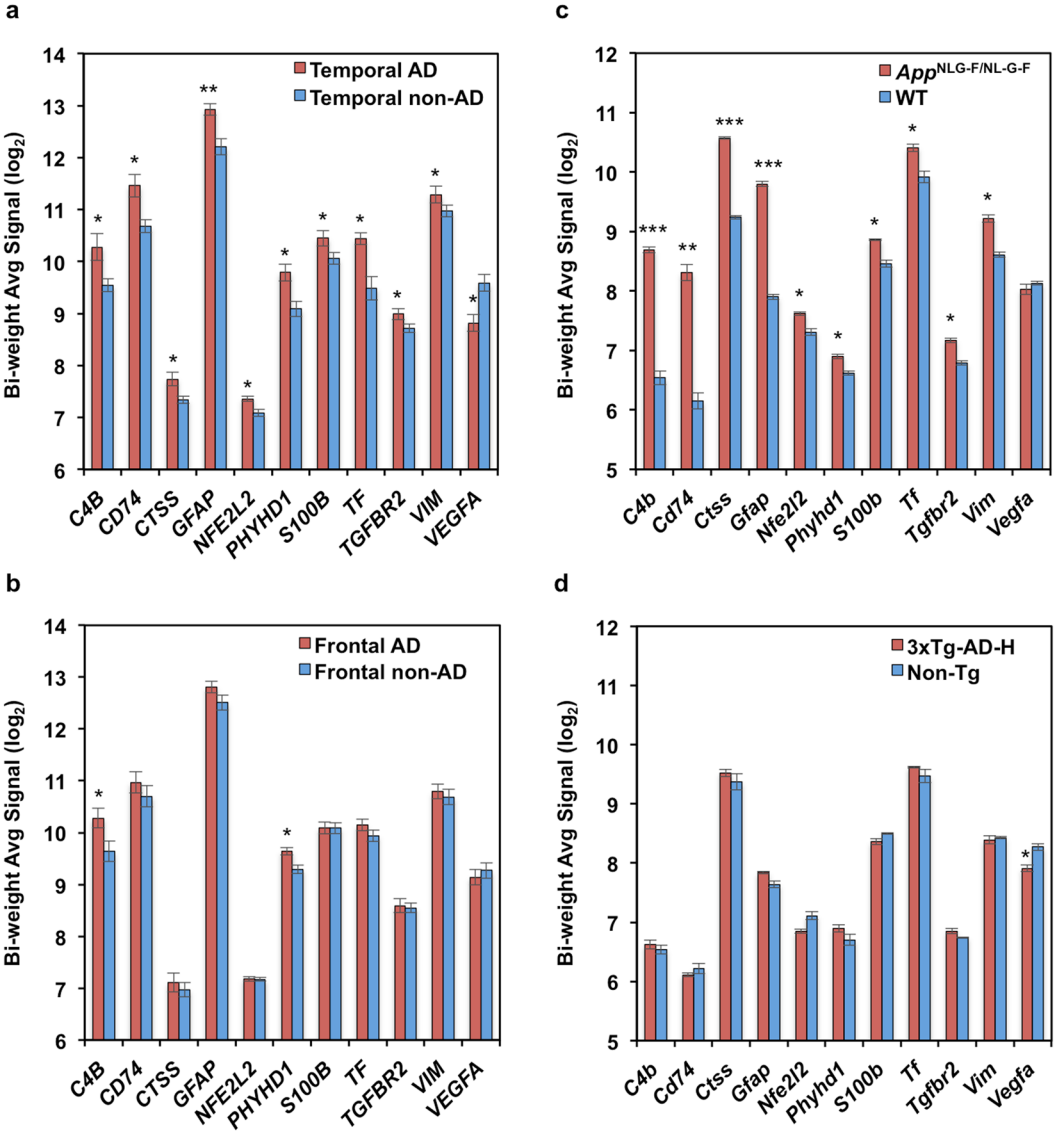
Comparison of expression levels of 11 common genes with functions related to Alzheimer’s disease. (**a**) Comparison between AD and non-AD temporal cortices. AD (n = 8), non-AD (n = 10). (**b**) Comparison between AD and non-AD frontal cortices. AD (n = 13), non-AD (n = 17). (**c**) Comparison between the *App* ^NL-G-F/NL-G-F^ and wild-type (WT) cortices (n = 3). (**d**) Comparison between the 3xTg-AD-H and non-Tg cortices (n = 3). Bi-weight average signal (log_2_) of the 11 AD-related genes obtained from microarray analysis are shown with SEM in bar graphs. Red bars, AD patients or AD mouse models; blue bars, non-AD or control mice. One-way between-subject ANOVA analysis was performed; *P < 0.05; **P < 0.001; ***P < 0.0001. Ten genes (*C4A*/*C4B*, *CD74*, *CTSS*, *GFAP*, *NFE2L2*, *PHYHD1*, *S100B*, *TF*, *TGFBR2* and *VIM*) were commonly upregulated in both human AD temporal and *App* ^NL-G-F/NL-G-F^ cortices.

One hundred genes (7.2%) among the 1372 altered genes in the AD temporal cortex, and 17 (7.2%) out of 236 altered genes in the AD frontal cortex were categorised as AD-related genes according to IPA function annotation, whereas in AD mouse models, a total of 37 out of 280 genes (13%) in the *App* ^NL-G-F/NL-G-F^ cortex, but only 2 out of 251 altered genes (0.8%) in 3xTg-AD-H cortex were categorised into the same group (Table 2). Among those genes, 10 genes (*C4A*/*C4B*, *CD74*, *CTSS*, *GFAP*, *NFE2L2*, *PHYHD1*, *S100B*, *TF*, *TGFBR2* and *VIM*) were commonly upregulated in the *App* ^NL-G-F/NL-G-F^ mouse and human AD temporal cortices (Fig. 4). In the human AD frontal cortex, *C4A*/*C4B* and *PHYHD1* genes were also significantly upregulated, and *CD74* and *GFAP* gene expression levels were increased (fold change: 1.20 and 1.23, respectively), but these increases were not statistically significant (Fig. 4).

**Table 2:** Genes significantly enriched in AD with significantly altered expression in the cortices of AD patients and AD mouse models.

Sample	p-value	# Genes	Genes
AD temporal	9.28E-08	100	*A2M*, *ABCG2*, *AGT*, *AKAP5*, *ALDH1L1*, *ALDH2*, *AQP1*, *ATP6V1E1*, *ATP6V1G2*, *BDNF*, *BGN*, *C3*, ***C4A/C4B***, *C5*, *CALB1*, ***CD74***, *CDK5*, *CHL1*, *CHRM1*, *CHRNB2*, *CNKSR2*, *CNKSR3*, *CNP*, *CTNNA3*, ***CTSS***, *CXCL12*, *CYP46A1*, *DLGAP2*, *DOK5*, *EEF2K*, *EPHA4*, *ETS2*, *FAM3C*, *FOLH1*, *FRMD4B*, *FRRS1*, *GABBR2*, *GABRA1*, *GABRA3*, *GABRA4*, *GABRA5*, *GABRB3*, *GABRG2*, ***GFAP***, *GLRB*, *GM2A*, *GRIA3*, *GRIN2A*, *GRIN2B*, *GRM5*, *HOMER1*, *HTR1A*, *HTR2A*, *INPP5A*, *IREB2*, *KLF3*, *LIPA*, *LY75*, *MAOB*, *MEGF10*, *MOG*, *MS4A4A*, *MS4A6A*, *MTR*, *NEFL*, *NFATC2*, ***NFE2L2***, *NPTX1*, *PAK1*, *PANK2*, ***PHYHD1***, *PIK3IP1*, *PLPPR4*, *PLTP*, *PON2*, *PRDX6*, *PRKCB*, *PRKCZ*, *PSEN1*, *PTPRE*, *QPCT*, *RAB6A*, *RIMS1*, *ROCK2*, ***S100B***, *SELENBP1*, *SEPP1*, *SLC2A1*, *SLC30A3*, *SNRNP70*, *SREBF1*, *SYNJ1*, *TAF13*, ***TF***, ***TGFBR2***, *THY1*, *TUBA8*, ***VEGFA***, ***VIM***, *VLDLR*
AD frontal	1.36E-02	17	*AGT*, *AKAP5*, *ATP6V1G2*, *BGN*, ***C4A/C4B***, *CALB1*, *CHRNB2*, *CYP46A1*, *DOK5*, *GABRA5*, *GAD2*, *GRIN2B*, ***PHYHD1***, *RAB6A*, *TAF13*, *TFRC*, *TSHZ2*
*App*^NL-G-F/NL-G-F^	1.51E-10	37	*Apoe*, ***C4a/C4b***, *Cd68*, ***Cd74***, *Ch25h*, *Cst3*, *Ctsb*, *Ctsd*, ***Ctss***, *Cyp11a1*, *F3*, *Fcgr1a*, *Fcgr2a*, *Fcgr2b*, ***Gfap***, *Hla-dqa1*, *Hspa1a/Hspa1b*, *Igf1*, *Lgmn*, *Lpl*, *Lrp10*, *Mag*, ***Nfe2l2***, *Pde3b*, ***Phyhd1***, *Pros1*, *Ptgs1*, *Rnaset2*, ***S100b***, *Serpina3*, *Sparc*, *Stat3*, ***Tf***, *Tgfb1*, ***Tgfbr2***, *Tyrobp*, ***Vim***
3xTg-AD-H	1.90E-03	2	***Vegfa***, *Xbp1*

Upregulated genes are underlined. Commonly upregulated genes in cortices of AD patients and *App* ^NL-G-F/NL-G-F^ mice are shown in bold underline. Commonly downregulated gene in cortices of AD patients and 3xTg-AD-H mice is shown in bold.

**Figure 5:**
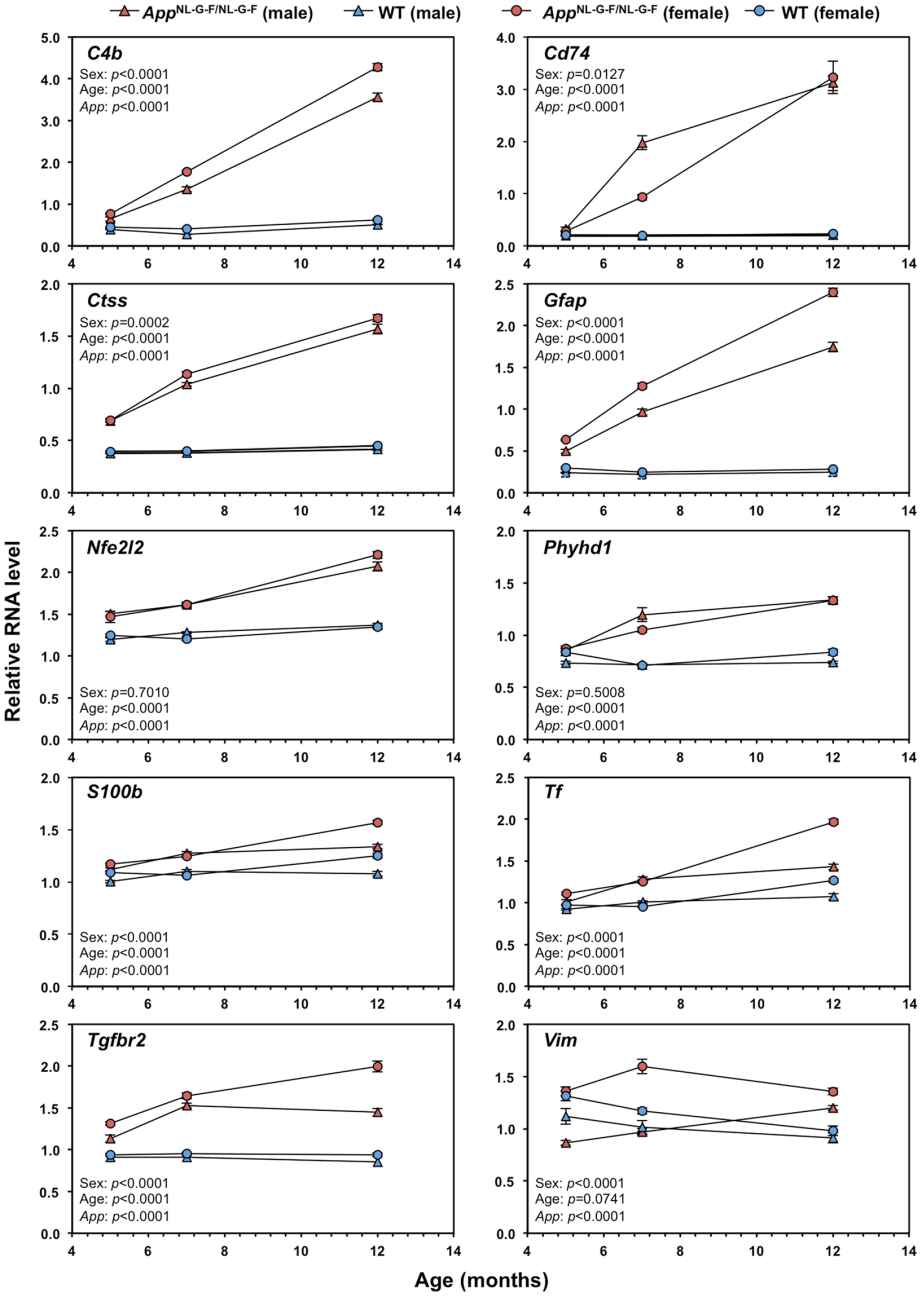
Effects of age and sex on expression levels of the 10 commonly upregulated genes related to Alzheimer’s disease in cortices of *App* ^NL-G-F/NL-G-F^ mice. Cortical RNA was isolated from male and female *App* ^NL-G-F/NL-G-F^ (red) and wild-type (WT, blue) mice (n = 3), at 5, 7 and 12 months of age, and subjected qRT-PCR. Expression levels, relative to *Gapdh*, of the 10 genes (*C4b*, *Cd74*, *Ctss*, *Gfap*, *Nfe2l2*, *Phyhd1*, *S100b*, *Tf*, *Tgfbr2* and *Vim*) commonly upregulated in both *App* ^NL-G-F/NL-G-F^ mouse and human AD temporal cortices are shown. Data is expressed as mean value ± SEM of three independent mice performed in triplicate. Three-way ANOVA was performed and *p*-values for effects (sex, age and *App* genotype [*App*]) are shown. Detailed results of statistical analysis are shown in Supplementary Figs S6 and S7.

We next evaluated expression levels of the 10 AD-related genes in the cortices of male and female *App* ^NL-G-F/NL-G-F^ and wild-type mice at 5, 7 and 12 months of age, in order to explore effects of gender and age on expression of the AD-related genes. During these periods, the area of Aβ deposition in the cortex progressively increases, together with behavioural symptoms starting at 8–9 months of age, and these events are more rapidly observed in female mice^10,11^. We performed qRT-PCR using RNA from entire cortex, and found that all 10 genes exhibited an age-dependent increase in their expression levels in male and female *App* ^NL-G-F/NL-G-F^ mice (Fig. 5, Supplementary Figs S6 and S7). At 5 months of age, there was no significant difference in expression levels of *Cd74*, *Phyhd1* (female), *Tf* (male) and *Vim* (female) genes between *App* ^NL-G-F/NL-G-F^ and wild-type mice, although expression of *C4b*, *Ctss*, *Gfap*, *Nef2l2*, *Phyhd1* (male), *S100b*, *Tf* (female), *Tgfbr2* and *Vim* (male) was significantly increased. In *App* ^NL-G-F/ NL-G-F^ mice, gene expression levels of *C4b*, *Ctss*, *Gfap*, *S100b*, *Tf*, *Tgfbr2* and *Vim* genes were greater in females than in males but *S100b*, *Tf* and *Tgfbr2* showed higher expression in females only at 12 months of age. At 7 months of age, male *App* ^NL-G-F/NL-G-F^ mice expressed higher levels of *Cd74* and *Phyhd1* than females, while there was no obvious gender difference in the expression level of *Nfe2l2*. Expression levels of *Vim* in female *App* ^NL-G-F/NL-G-F^ mice were higher than in males at any age, reaching its peak at 7 months of age, then decreasing, while males exhibited a continuous increase during aging. In the human AD brain, expression levels of *PHYHD1* in the AD frontal cortex were significantly greater in females than males (ANOVA: *P* = 0.0097), and *VIM* expression in the AD temporal cortex was greater in females (ANOVA: *P = *0.0697).

**Figure 6:**
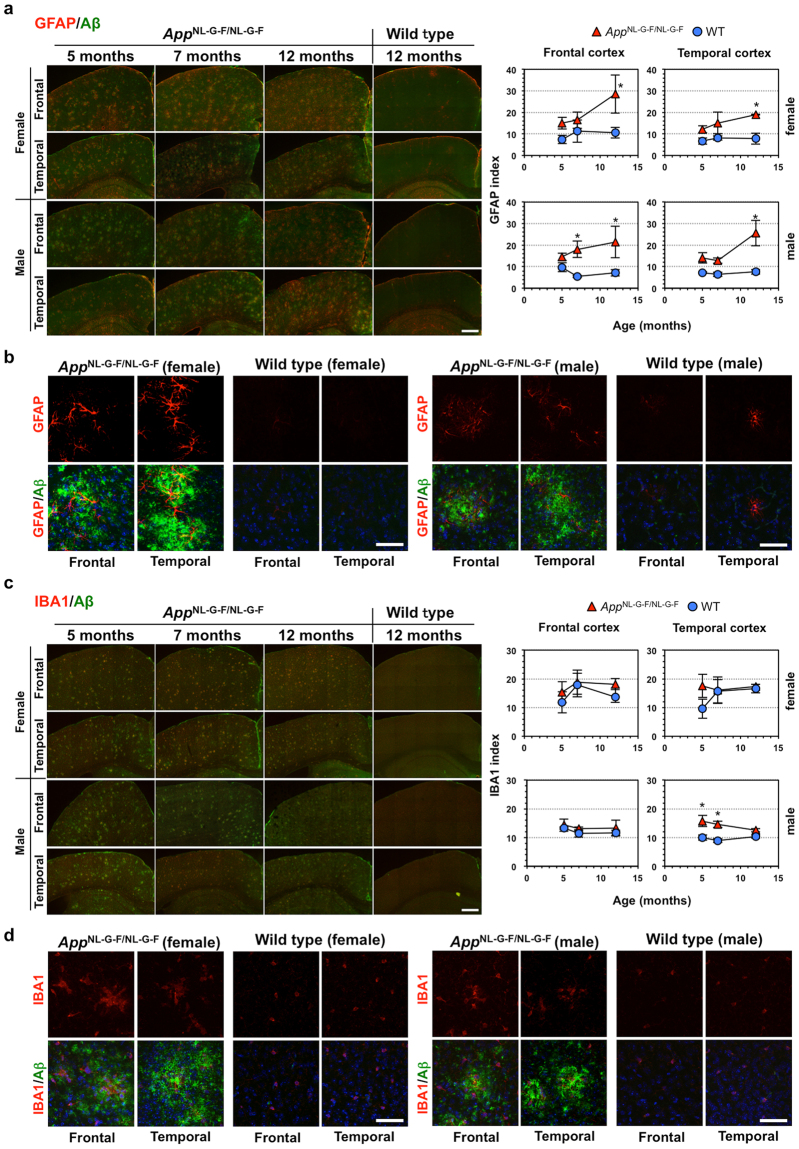
Effects of age and sex on amyloid β deposition and glial activation in cortices of *App* ^NL-G-F/NL-G-F^ mice. Double-immunofluorescence microscopy for Aβ and GFAP (**a**,**b**), and Aβ and IBA1 (**c**,**d**). Coronal sections containing frontal (Bregma: −1.255 to −1.455) and temporal (Bregma: +1.845 to +2.045) cortices prepared from 5-, 7- and 12-month-old, male and female *App* ^NL-G-F/NL-G-F^ and wild-type (WT) mice, were subjected to double-immunofluorescence microscopy using mouse anti-human Aβ (green) and either anti-GFAP or rabbit anti-IBA1 antibodies (red). (**a**,**c**) Multiple z-stack images of 15 fields were tiled and stacked together using ZEN imaging software. Each immunoreactivity was measured, and means with SEM (n = 3) of the GFAP (**a**) and IBA1 (**c**) index are shown in the graphs on the right. Scale bar = 500 μm. Student’s *t*-test was performed between the two mouse lines at the given ages; *P < 0.05. (**b**,**d**) Magnified images for 5-month-old samples are shown. Nuclei were stained with DAPI (blue) in double-immunofluorescence images. Scale bar = 50 µm.

Finally, we performed double-immunofluorescence microscopy for Aβ and GFAP or Aβ and IBA1 using frontal and temporal cortices prepared from 5-, 7- and 12-month-old, male and female *App* ^NL-G-F/NL-G-F^ mice, in comparison with wild-type mice (Fig. 6). We confirmed significant Aβ deposition in *App* ^NL-G-F/NL-G-F^ but not wild-type cortex as early as at 5 months of age, as we reported previously^10,11^.

Weak GFAP immunoreactivity was heterogeneously distributed and mainly restricted to subcortical and hippocampal areas in the wild-type brain, while in the *App* ^NL-G-F/NL-G-F^ brains, astrocytes with strong GFAP immunoreactivity were detected in areas surrounding Aβ plaques in the cortex as early as at 5 months of age, and the levels of immunoreactivity increased during aging (Fig. 6a,b).

IBA1 immunoreactivity was detected in all brain regions in both wild-type and *App* ^NL-G-F/NL-G-F^ mice and the levels of immunoreactivity were not altered much during aging. However, in the *App* ^NL-G-F/NL-G-F^ brains, morphologically activated microglia were highly clustered inside Aβ plaques as early as at 5 months of age (Fig. 6c,d).

We also performed double-immunofluorescence microscopy for Aβ and GFAP or Aβ and IBA1 using frontal and temporal cortices prepared from 7- and 12-month-old, male and female 3xTg-AD-H mice, in comparison with non-Tg mice (Supplementary Figs S8 and S9). Considerably weaker immunoreactivities for Aβ, GFAP and IBA1 were detected in the 3xTg-AD-H brain compared with the *App* ^NL-G-F/NL-G-F^ brain. In 3x Tg-AD-H cortex, Aβ immunoreactivity, which became more apparent at 12 months of age, was detected in the deep cortical layers, mostly within the cell bodies or neuropil (Supplementary Fig. S10), indicating intracellular accumulation of Aβ. Astrocytes with strong GFAP immunoreactivity were detected in areas surrounding Aβ-positive cells in the cortex (Supplementary Fig. S8a,b), while distribution and morphology of the IBA1-positive microglia were similar between the two mouse strains from 7 to 12 months of age.

Taken together, the different profiles of gene expression between *App* ^NL-G-F/NL-G-F^ and 3xTg-AD-H cortices reflect the extents of gliosis, inflammatory responses and Aβ pathology.

### *App*^**NL-G-F/NL-G-F**^ cortex exhibits altered expression of genes defined as risk factors for AD by the genome-wide association study together with genes in the immune/microglia module

Finally, we evaluated the expression levels of 57 genes defined as risk factors for AD by genome-wide association study (GWAS), together with genes in the immune/microglia module (*CTSC*, *DOCK2*, *FCER1G*, *HCK*, *LY86*, *S100A11*, and *TYROBP*) whose expression is reported to be significantly altered in late-onset AD (LOAD) patients and *APP* ^K670N/M671L^/*PSEN1* ^M146V^ transgenic mice^23–30^, in our microarray data from human and mouse brains (Supplementary Table S10). As shown in Table 3, expression levels of four genes (*DOCK2*, *INPP5D*, *LY86*, and *PSEN1*) were significantly increased in human AD temporal but not frontal cortex, while expression of *GRIN2B* was significantly decreased in both human AD cortices. In *App* ^NL-G-F/NL-G-F^ cortex, expression levels of 13 genes (*Abi3*, *Apoe*, *Bin2*, *Cd33*, *Ctsc*, *Dock2*, *Fcer1g*, *Hck*, *Inpp5D*, *Ly86*, *Plcg2*, *Trem2*, and *Tyrobp*) were significantly increased, and that of *Frmd6* was significantly decreased. In 3xTg-AD-H cortex, expression levels of two genes (*Ab13* and *Frmd6*) were significantly decreased, and only that of *Trem2* was significantly increased.

**Table 3:** Expression of genes defined as risk factors for AD by GWAS and the immune/microglia module in cortices of AD patients and AD mouse models.

Gene Symbol	Fold change (relative to non-AD control)				References
	AD temporal	AD frontal	*App*^NL-G-F/NL-G-F^	3xTg-AD-H	
*ABI3*	1.06	1.05	**1**.**34**	**−1**.**20**	^23^
*APOE*	−1.04	1.07	**1**.**23**	−1.01	^24–27^
*BIN2*	1.22	1.04	**1**.**28**	1.04	^28^
*CD33*	1.13	1.11	**1**.**20**	−1.05	^24–27,29^
*FRMD6*	−1.06	−1.06	**−1**.**22**	**−1**.**26**	^26^
*GRIN2B*	**−1**.**42**	**−1**.**26**	−1.11	−1.04	^28^
*INPP5D*	**1**.**33**	1.14	**1**.**60**	1.15	^24,26,27,29,30^
*PLCG2*	1.19	1.03	**1**.**26**	1.02	^23^
*PSEN1*	**1**.**23**	−1.02	1.00	−1.18	^24^
*TREM2*	1.17	1.22	**3**.**42**	**1**.**41**	^23,24,26,27^
*CTSC*	1.28	1.06	**1**.**69**	−1.23	^25^^*^
*DOCK2*	**1**.**44**	1.27	**1**.**50**	1.06	^25^^*^
*FCER1G*	1.17	1.13	**2**.**28**	1.03	^25^^*^
*HCK*	1.03	−1.01	**1**.**34**	1.08	^25^^*^
*LY86*	**1**.**53**	1.15	**3**.**17**	−1.01	^25^^*^
*TYROBP*	1.26	1.12	**3**.**42**	1.04	^25^^*^

Fold changes of significantly altered genes between AD vs. non-AD or AD mouse model vs. control (ANOVA: *P* < 0.05, fold change ≥ 1.2 or ≤−1.2) are shown in bold. Results of 57 genes examined are shown in Supplementary Table S10.

*Genes differentially expressed in prefrontal cortex among control, MCI, and LOAD samples.

Taken together, our results indicate that expression of genes defined as risk factors for AD by GWAS, together with genes in the immune/microglia module, was predominantly increased in *App* ^NL-G-F/NL-G-F^ mice, as observed in LOAD patients and in *APP* ^K670N/M671L^/*PSEN1* ^M146V^ transgenic mice^23–30^.

## Discussion

In the present study, we performed inter-species comparative gene expression profiling using cortical RNA prepared from AD patient brains (frontal and temporal cortices) and two different AD mouse models (*App* ^NL-G-F/NL-G-F^ and 3xTg-AD-H). The AD patient brains exhibited a much larger number of genes with altered expression in temporal cortex than in frontal cortex. Expression levels of 59 genes were commonly altered in the *App* ^NL-G-F/NL-G-F^ and human AD temporal cortices, and most of these genes (34 genes) were related to inflammatory response or immunological disease. Among them, expression of 10 genes (*C4A*/*C4B*, *CD74*, *CTSS*, *GFAP*, *NFE2L2*, *PHYHD1*, *S100B*, *TF*, *TGFBR2* and *VIM*), which are categorised as AD-related by IPA, was increased in the *App* ^NL-G-F/NL-G-F^ cortex as Aβ amyloidosis progressed with exacerbated neuroinflammation. Only 17 genes were commonly altered in the 3xTg-AD-H and human AD temporal cortices, most of which related to neurological disease.

In human AD, only the temporal cortex exhibited significant upregulation of several marker genes for astrocytes, microglia and oligodendrocytes, and significant downregulation of several neuronal marker genes (Table 1), supporting results showing that the AD temporal cortex generally exhibits more rapid progression of AD pathologies, including neuronal loss, than frontal cortex^31–33^. Contrary reports have shown more significant reduction in the thickness of frontal cortex than temporal cortex, and yet an effect of brain inflammation cannot be excluded^12,14^. When we compared *App* ^NL-G-F/NL-G-F^ and 3xTg-AD-H cortices, we noticed that the two AD mouse models exhibited different gene expression profiles (Fig. 1e). It is noteworthy that only the *App* ^NL-G-F/NL-G-F^ cortex exhibited a significant upregulation of several marker genes for astrocytes, microglia and oligodendrocytes, similar to human AD temporal cortex (Table 1). These results suggest that expression changes in the *App* ^NL-G-F/NL-G-F^ cortex correlate with pathological features observed in human AD temporal cortex. The 3xTg-AD-H cortex shared a total of 20 genes (*Abhd6*, *Cyth3*, *Cckbr*, *Dusp6*, *Egr3*, *Fndc5*, *Gramd4*, *Homer1*, *Kcnf1*, *Klf10*, *Mkl1*, *Nab2*, *Nptx2*, *Pcsk1*, *Qpct*, *Tet3*, *Tipin*, *Trub2*, *Ttpal* and *Vegfa*) with the human AD hippocampus^18^, some of which are related to neuronal metabolic and synaptic functions, sugesting that the 3xTg-AD-H cortex mimics hippocampal and to lesser extent cortical profiles in AD patient brains. These results support the fact that different AD mouse models represent different features of human AD pathologies^7^.

As expected from the gene expression profiles, *App* ^NL-G-F/NL-G-F^ mice exhibit aggressive extracellular Aβ deposition as early as at 5 months of age, and gliosis from 7 to 12 months of age, throughout cortical and hippocampal regions, and memory impairment in an age-dependent manner (Fig. 6)^10,11^. The 3xTg-AD-H mice exhibit mainly intracellular Aβ accumulation before 12 months of age, accompanied by increased levels of intracellular APP sub-products, as well as Tau pathologies such as intracellular NFT and cognitive impairment, accompanied by astrocytosis but not microgliosis (Supplementary Figs S8, S9 and S10)^9,17,34,35^. Differences in both the gene expression profiles and pathologies observed between the two AD mouse models strongly suggest that extracellular but not intracellular Aβ induces gliosis, namely neuroinflammatory responses, similar to what is observed in human AD temporal cortex. In the comparison of the human cortex data to 3xTg-AD-H cortex, two genes (*Pcsk1* and *Vegfa*) were categorized to inflammatory response or immunological disease (Fig. 2b), but these genes were not altered in the *App* ^NL-G-F/NL-G-F^ cortex. In contrast, intracellular accumulation of Aβ and other APP sub-products, and/or Tau pathologies, are likely related to the neuronal metabolic and synaptic dysfunctions, as evident in the hippocampus of both human AD and 3xTg-AD-H brains^9,18^.

Some neuronal marker genes in human AD cortex were significantly downregulated, in accordance with the neuronal loss in human AD cortex^16,31–33,36^. In contrast there was no downregulation of neuronal marker genes in cortices from the two AD mouse models (Table 1), both of which do not exhibit neuronal loss^7,10,17^, thus indicating that the different profiles of gene expression detected between the two mouse models were not due to neuronal loss. We note that several genes involved in neuronal function, such as *Egr3*, *Egr4*, *Fosl2*, *Grik1*, *Homer1*, *Lig4*, *Npas4*, *Nptx1*, *Pcsk1*, *Vegfa* and *Xbp1* were downregulated, especially in the 3xTg-AD-H cortex (Supplementary Tables S6 and S7), which may correlate with the previously reported cognitive impairment^17,18^.

The human AD temporal cortex exhibits significantly altered expression of 100 AD-related genes, while only 17 genes were altered in AD frontal cortex. The *App* ^NL-G-F/NL-G-F^ cortex also exhibited significantly altered expression of 37 AD-related genes; 10 of these genes were in common with human AD temporal cortex, and two genes were in common with AD frontal cortex. There were only two AD-related genes altered in the 3xTg-AD-H cortex (Table 2). These results suggest that Aβ amyloidosis alone causes changes in gene expression profiles in the cortex, especially in the temporal cortex. Immunofluorescence microscopy revealed that the *App* ^NL-G-F/NL-G-F^ mice exhibited progressive Aβ deposition and microgliosis, with similar extents in the frontal and temporal cortices. The astrocytosis progression was likely to be greater in female *App* ^NL-G-F/NL-G-F^ mice, and the two cortical regions tended to respond differently to Aβ amyloidosis (Figs 5,6). This may also be the case in human AD brain, which could explain why gene expression profiles were different between the temporal and frontal cortices in AD patients.

Studies on post-mortem brains have shown that AD pathologies are accompanied by neuroinflammation, probably as a consequence of Aβ amyloidosis or neuronal damage. However, recent neuroimaging and genome-wide association studies further suggest that neuroinflammation is an early event that takes place even before Aβ amyloidosis^4,6,13^. In the present study, we showed that microgliosis and/or astrocytosis was progressively apparent with the progression of Aβ amyloidosis in the *App* ^NL-G-F/NL-G-F^ cortex, and these pathological events were accompanied by progressively increased expression of genes involved in inflammatory responses, such as *C4b*, *Cd74*, *Ctss*, *Gfap*, *Nfe2l2*, *S100b*, *Tf*, *Tgfbr2* and *Vim*, which constituted three functional networks (Figs 3,5 and 6). In these networks, the expression of 48 genes, including the 10 AD-related genes, was commonly altered in the *App* ^NL-G-F/NL-G-F^ mouse and human AD temporal cortices. Among these genes, expression of *C4*, *Mpeg1*, *Lilrb4*, *Slc14a1*, *Ctsh*, *B2m*, *Aif1* and *Ly86* has been shown to be upregulated in astrocytes and/or microglia in the double-transgenic *APP*swe/*PS1*dE9 mouse frontal cortex^25^. Network-based integrative analysis of genetic risk loci for LOAD identified by GWAS have revealed the immune/microglia module as the molecular system most strongly associated with the pathophysiology of LOAD, and also identified the key network regulators, including TYROBP, which are upregulated in LOAD^25^. Moreover, a genome-wide gene-expression analysis in wild-type and five transgenic mouse lines with only Aβ (*APP* ^K670N/M671L^; *PSEN1* ^M146V^; hemizygous and homozygous *APP* ^K670N/M671L^/*PSEN1* ^M146V^) or only Tau (*MAPT* ^P301L^) pathology revealed that immune gene expression correlated tightly with Aβ plaques, whereas synaptic genes correlated negatively with NFTs^26^.

When we examined the expression levels of 57 genes defined as risk factors for AD by GWAS, together with genes in the immune/microglia module^23–30^, in our microarray data from human and mouse brains (Table 3, Supplementary Table S10), we found that 13 genes (*Abi3*, *Apoe*, *Bin2*, *Cd33*, *Ctsc*, *Dock2*, *Fcer1g*, *Hck*, *Inpp5D*, *Ly86*, *Plcg2*, *Trem2*, and *Tyrobp*) were significantly upregulated in the *App* ^NL-G-F/NL-G-F^ cortex. Among these, overexpression of TYROBP in microglial cells has been reported to alter the expression of the microglia module that is dominated by genes involved in pathogen phagocytosis^25^. Moreover, *Fcer1g* and *Trem2* have been identified as member of the hub genes (*C1qa*, *C1qb*, *Fcer1g*, *Trem2*, and *Tlr2*) of the immune module in the cortex from transgenic mouse lines with only Aβ pathology but not with only Tau pathology^26^. Our results clearly indicate that the prominent neuroinflammation observed in the *App* ^NL-G-F/NL-G-F^ cortex is a result of pure Aβ pathology induced by *App* knock-in mutations. Only the *Trem2* gene was mildly upregulated in 3xTg-AD-H cortex, in agreement with their milder Aβ pathology and inflammatory responses in comparison with *App* ^NL-G-F/NL-G-F^ cortex (Supplementary Figs S8 and S9). The present study indicates that Aβ pathology caused by authentic expression of the pathogenic Aβ in *App* ^NL-G-F/NL-G-F^ mice predominantly activates the immune-specific module, as observed in LOAD patients and in *APP* ^K670N/M671L^/*PSEN1* ^M146V^ transgenic mice^23–30^.

Expression of several complement component genes (*C1*, *C3*, *C4*, *C5*) was significantly increased in the *App* ^NL-G-F/NL-G-F^ and human AD cortices. A recent report showed a significant increase in the copy number of *C4* genes in AD patients, compared with healthy controls^37^, which may contribute to the elevated levels of C4 in cerebrospinal fluid or serum in the AD patients^38,39^. Moreover, it has been shown that C4 surrounds Aβ plaques in the cortex of an AD mouse model^40^. C4b, a cleaved product of C4 by the C1 complex, which can be activated by Aβ^41^, functions as a C3 convertase with C2b, thus resulting in activation of the complement system, which may in turn inappropriately activate microglia, thereby mediating synapse loss^42^ or a further inflammatory response^43^.

*CD74*, the expression of which was also significantly increased in the *App*^NL-G-F/NL-G-F^ and human AD cortices, encodes an integral membrane protein that acts as a chaperone for MHC class II molecules and a receptor binding site for macrophage migration inhibitory factor (MIF)^44^. It has been shown that CD74 expression increases in microglia, astrocytes and NFT-positive neurons of AD patients^45,46^. Moreover, CD74 was reported to interact with APP and suppress production of Aβ^47,48^, while CD74 itself is processed by cathepsin S, encoded by *CTSS*, thus releasing its cytoplasmic domain^49^, which is essential for the proinflammatory NF- κB activation^50^. Because only full-length CD74 can interact with APP^48^, an increased expression of cathepsin S in the AD cortex could deplete the full-length CD74, thereby cancelling the suppression of Aβ production and rather activating NF-κB. Additionally, cathepsin S could be involved in lysosomal processing of APP to produce Aβ^51^.

In the *App* ^NL-G-F/NL-G-F^ cortex, gene expression of *Ctss* as well as *Nfe2l2*, *Tgfbr2* and *Gfap* was already elevated at 5 months of age compared with wild-type mice (Fig. 5), suggesting its contribution in Aβ production at the early stage of AD development. Conversely, increased gene expression of *Cd74*, *C4b*, *Phyhd1*, *Tf* and *S100b* was detected at 7 months of age, suggesting that expression of these genes may require higher levels of Aβ accumulation.

When we compared expression levels of the 10 genes shown in Fig. 5, *C4b*, *Ctss*, *Gfap*, *S100b*, *Tf*, *Tgfbr2* and *Vim* exhibited significantly higher expression in female *App* ^NL-G-F/NL-G-F^ mice. It has been hypothesised that sex hormones, such as oestrogen and androgen play important roles in aging that are linked to sex vulnerability and to AD^52,53^. As seen in Network 3 (Fig. 3), expression of *VIM* was reported to be partly dependent on oestrogen receptor β (ERβ)^54^, which shows decreased levels with age but remains responsive to oestradiol treatment^55^. This may explain why the female-specific *Vim* expression in *App* ^NL-G-F/NL-G-F^ cortex peaked at 7 months of age (Fig. 5). *Tgfbr2*, increased in the female *App* ^NL-G-F/NL-G-F^ cortex during aging, is also involved in oestrogen responses^56^. Thus, oestrogen and TGF-β may play roles in the female-specific expression of genes encoding cytoskeletal proteins (GFAP, S100B, and/or VIM), in complex manners^57–59^. Our results thus suggest that oestrogen together with TGF-β may induce more severe Aβ amyloidosis and changes in gene expression profiles in females.

In conclusion, the *App* ^NL-G-F/NL-G-F^ mouse, a novel AD mouse model with authentic expression of pathogenic Aβ, exhibits a cortical gene expression profile that reproduces changes observed in the human AD brain, in a limited but faithful manner. Results from the present study indicate a strong correlation between cortical Aβ amyloidosis and neuroinflammation, and also provide important clues to better understand the role of gender effects in AD development.

## Methods

### Ethics statement

The use of human postmortem brain tissue was approved by the Ethics Committee of the Faculty of Medicine, Kyushu University, Fukuoka, Japan, and was performed in accordance with the ethical standards described in the latest revision of the Declaration of Helsinki. Written informed consent for all subjects was obtained from their families. The handling and killing of all animals was performed in accordance with national prescribed guidelines, and ethical approval for the study was granted by the Animal Experiment Committee of Kyushu University, Fukuoka, Japan.

### Total RNA prepared from post-mortem brain tissues

We previously prepared total RNA from freshly frozen cerebral cortices of frontal and temporal poles at several centimeters thick removed from post-mortem brains donated for the Hisayama study between December 15, 2008 and February 24, 2011^18^. All RNA samples were preserved at −80 °C until further use.

### Analysis of human microarray data

Previously obtained microarray data using total RNA prepared from human temporal (10 AD, 19 non-AD cases) and frontal cortices (15 AD and 18- non-AD cases)^18^ are available from the GEO database (accession number GSE36980). CEL files were imported into the Affymetrix Expression Console (Affymetrix Japan K.K., Tokyo, Japan) and CHP files were obtained using a Gene Level-RMA-Sketch method. CHP files were input into the Affymetrix Transcriptome Analysis Console (TAC) software and a gene level differential expression analysis was performed according to the user’s guide. One-way between subject ANOVA was performed between AD and non-AD subjects and a list of transcripts was created. Principal Component Analysis (PCA) and hierarchical clustering was performed in the Affymetrix Expression Console and TAC software, respectively, and several samples were found to be outliers, likely owing to biological heterogeneity or technical issues (Supplementary Figs S1 and S2). These outliers were excluded to avoid undesirable artefacts during the profiling analyses.

### Animals

The homozygous triple-transgenic mouse model of AD (3xTg-AD-H), carrying a homozygous *Psen1*_M146V_ knock-in mutation and homozygous mutant transgenes for Swedish *APP*_KM670/671NL_ and *MAPT*_P301L_, and control non-Tg mice were previously established^17,18^. Heterozygous *App* ^+/NL-G-F^ mice carrying humanised Aβ sequence (G601R, F606Y, R609H), Swedish (ML595/596NL), Beyreuther/Iberian (I641F), and Arctic (E618G) mutations, were previously established^10^. Homozygous *App* ^NL-G-F/NL-G-F^ and wild-type mice were obtained by crossing, and were maintained as inbred lines. All animals were maintained in a specific pathogen-free room.

### Mouse brain tissue preparation

For transcriptomic analyses, mice were anesthetized, transcardially perfused with saline, and brain cortices were quickly dissected, snap-frozen in liquid nitrogen, and preserved at −80 °C until RNA preparation. For immunofluorescence, mice were perfused with saline followed by cold 4% paraformaldehyde (PFA) in phosphate-buffered saline (PBS). The brains were removed and post-fixed in 4% PFA for 24 hours at 4 °C. Tissue blocks were cryoprotected in 20% sucrose, followed by 30% sucrose, in PBS, and then embedded in FSC 22 frozen section media (Leica Microsystems K.K, Tokyo Japan). The tissue blocks were quickly frozen and stored at −80 °C until further use.

### RNA isolation and microarray analysis

We performed microarray analyses using cortical RNA prepared from 12-month-old *App* ^NL-G-F/NL-G-F^ and 3xTg-AD-H mice together with the corresponding wild-type or non-Tg control mice, respectively (3 males for each group). Total RNA was prepared from frozen cortex using Isogen (Nippon Gene, Tokyo, Japan) according to the manufacturer instructions. RNA concentrations were determined by measuring the UV absorbance spectra, and the total RNA profile was analysed using an Agilent 2100 Bioanalyzer (Agilent Technologies Japan, Tokyo, Japan) to determine RNA integrity number (RIN). RNA (100 ng) was used for microarray analysis. The GeneChip WT PLUS Reagent Kit (Affymetrix Japan K.K.) was used to generate amplified and biotinylated sense-strand DNA targets. Manufacturer instructions were followed for hybridisation, washing, and scanning steps with Affymetrix Mouse Gene 2.0ST Array, and CEL files were generated. CEL files were further analysed as described for analysis of human microarray data. The lists of transcript clusters significantly altered (ANOVA: *P* < 0.05, fold change ≥ 1.2 or ≤−1.2, bi-weight average signal (log_2_) > 6.64, compared with control) were further analysed using Ingenuity Pathway Analysis (IPA, Tomy Digital Biology Co., Ltd., Tokyo, Japan) software to determine the commonly altered genes between AD patients and each AD mouse model, as well as the relevant biological function categories and network-based interactions. All microarray data were deposited in the GEO database (accession number GSE92926).

### Reverse transcription and quantitative polymerase chain reaction

RNA samples were reverse-transcribed to first-strand cDNA using 1 μg of total RNA, random primers, and the High-Capacity cDNA Reverse-Transcription Kit (Life Technologies Japan Ltd., Tokyo, Japan). Primer pairs (listed in Supplementary Table S3) and cDNA dilutions were optimised for real-time quantitative reverse-transcription PCR (qRT-PCR) using Thermal Cycler Dice® Real-Time System Single (Takara Bio Inc., Kusatsu, Japan). For each qRT-PCR, 0.5% of the total cDNA yield was used, in triplicates. Relative expression levels of each gene were obtained using the 2nd Derivative Maximum (SDM) standard curve method^60^. *Gapdh* was used as an internal control and we verified that *Gapdh* levels do not change between mutant and control mice (Supplementary Fig. S11).

### Double-immunofluorescence microscopy

Serial coronal sections (40 μm thickness) were prepared using a cryostat and collected as free-floating sections. Sections were blocked in 2 × Block Ace solution (Dainippon Pharmaceutical, Osaka, Japan) for 2 hours at room temperature, then incubated with a corresponding mix of primary antibodies (mouse anti-human Aβ 82E1 (10323; 1:4000; IBL Japan), and either rabbit anti-GFAP (Z0334; 1:2000; Dako Japan Inc., Kyoto, Japan) or anti-IBA1 (019–19741, 1:500, Wako Pure Chemical Industries Ltd., Osaka, Japan)) overnight at 4 °C. Corresponding Alexa Fluor-labelled secondary antibodies (Life Technologies Japan) were then added and incubated for 45 minutes at room temperature, followed by 0.05 μg/ml DAPI for 10 min at room temperature, and mounted on slides. All sections were rinsed in 0.3% Triton X-100 in PBS, 3 times for 5 min. The sections were mounted on glass slides and air-dried. The sections were then embedded with VECTASHIELD Mounting Medium (Vector Laboratories, Ltd., Burlingame, CA, USA). Multiple z-stack images of 15 fields were obtained, tiled, and stacked together using a confocal microscope (LSM700, Carl Zeiss Microscopy, Tokyo, Japan) with Zen 2012 software (Carl Zeiss Microscopy). The intensity of GFAP or IBA1 immunofluorescence was measured in each digital image using ImageJ 1.51n (NIH) to obtain the GFAP or IBA1 index, which corresponds to one thousandth of the mean intensity per µm^2^.

### Statistical analysis

Gene-level estimates from microarray data were subjected to one-way between subject ANOVA using Affymetrix TAC software. Statistical analysis was performed using JMP Pro Version 13.2.0 software (SAS Institute, Raleigh, NC, USA). A *P*-value < 0.05 was considered statistically significant.

## Electronic supplementary material

Supplementary Information

Supplementary Table S1

Supplementary Table S2

Supplementary Table S3

Supplementary Table S4

Supplementary Table S5

Supplementary Table S6

Supplementary Table S7

Supplementary Table S8

Supplementary Table S9

Supplementary Table S10
