# Author Correction: Comparative profiling of cortical gene expression in Alzheimer’s disease patients and mouse models demonstrates a link between amyloidosis and neuroinflammation

**DOI:** 10.1038/s41598-021-97918-9

**Published:** 2021-09-09

**Authors:** Erika Castillo, Julio Leon, Guianfranco Mazzei, Nona Abolhassani, Naoki Haruyama, Takashi Saito, Takaomi Saido, Masaaki Hokama, Toru Iwaki, Tomoyuki Ohara, Toshiharu Ninomiya, Yutaka Kiyohara, Kunihiko Sakumi, Frank M. LaFerla, Yusaku Nakabeppu

**Affiliations:** 1 Division of Neurofunctional Genomics, Department of Immunobiology and Neuroscience, Medical Institute of Bioregulation, Kyushu University, 3-1-1 Maidashi, Higashi-ku, Fukuoka, 812–8582 Japan; 2 Laboratory for Proteolytic Neuroscience, RIKEN Brain Science Institute, Saitama, Japan; 3 Department of Neuropathology, Neurological Institute, Graduate School of Medical Sciences, Kyushu University, 3-1-1 Maidashi, Higashi-ku, Fukuoka, 812–8582 Japan; 4 Department of Neuropsychiatry, Graduate School of Medical Sciences, Kyushu University, 3-1-1 Maidashi, Higashi-ku, Fukuoka, 812–8582 Japan; 5 Department of Epidemiology and Public Health, Graduate School of Medical Sciences, Kyushu University, 3-1-1 Maidashi, Higashi-ku, Fukuoka, 812–8582 Japan; 6 Hisayama Research Institute for Lifestyle Diseases, Hisayama, Fukuoka Japan; 7 Department of Neurobiology and Behavior, University of California, Irvine, CA 92697 USA; 8 Present Address: Department of Neurology, University of California, San Francisco, San Francisco, CA 94158 USA; 9 Department of Neurosurgery, Japan Community Health Care Organization Kyushu Hospital, Kitakyushu, 806–8501 Japan

Correction to: *Scientific Reports* 10.1038/s41598-017-17999-3, published online 19 December 2017

This article contains errors in the Introduction, where

“*App*^NL-G-F/NL-G-F^ mice carrying the homozygous mutant *App* gene encoding the humanised Aβ sequence (G601R, F606Y, and R609H) with three pathogenic mutations, namely Swedish (KM595/596NL), Beyreuther/Iberian (I641F), and Arctic (E618G)^10^, progressively exhibit Aβ accumulation starting at 4 to 6 months of age, dense distributions of microglia and astrocytes from 9 months of age, and behavioural symptoms from 8 to 12 months of age^10,11^.”

should read:

“*App*^NL-G-F/NL-G-F^ mice carrying the homozygous mutant *App* gene encoding the humanised Aβ sequence (G676R, F681Y, and R684H) with three pathogenic mutations, namely Swedish (KM670/671NL), Beyreuther/Iberian (I716F), and Arctic (E693G)^10^, progressively exhibit Aβ accumulation starting at 4 to 6 months of age, dense distributions of microglia and astrocytes from 9 months of age, and behavioural symptoms from 8 to 12 months of age^10,11^.”

In addition, in the Methods section, under the subheading ‘Animals’,

“Heterozygous *App*^+/NL-G-F^ mice carrying humanised Aβ sequence (G601R, F606Y, R609H), Swedish (ML595/596NL), Beyreuther/Iberian (I641F), and Arctic (E618G) mutations, were previously established^10^.”

should read:

“Heterozygous *App*^+/NL-G-F^ mice carrying humanised Aβ sequence (G676R, F681Y, R684H), Swedish (KM670/671NL), Beyreuther/Iberian (I716F), and Arctic (E693G) mutations, were previously established^10^.”

