# Supplementary material for: Comparative profiling of cortical gene expression in Alzheimer’s disease patients and mouse models demonstrates a link between amyloidosis and neuroinflammation
Source: Sci Rep. 2017 Dec 19;7:17762. doi: 10.1038/s41598-017-17999-3 (PMC5736730; doi:10.1038/s41598-017-17999-3)
Supplement: Supplementary file 1 — Supplementary Information [file 41598_2017_17999_MOESM1_ESM.pdf]

Supplementary Information for:

Comparative profiling of cortical gene expression in Alzheimer's disease patients and mouse models demonstrates a link between amyloidosis and neuroinflammation

Erika Castillo[1], Julio Leon[1,†], Guianfranco Mazzei[1], Nona Abolhassani[1], Naoki Haruyama[1], Takashi Saito[2], Takaomi Saido[2], Masaaki Hokama[1,§], Toru Iwaki[3], Tomoyuki Ohara[4], Toshiharu Ninomiya[5], Yutaka Kiyohara[6], Kunihiko Sakumi[1], Frank M. LaFerla[7], and Yusaku Nakabeppu[1].

[1]Division of Neurofunctional Genomics, Department of Immunobiology and Neuroscience, Medical Institute of Bioregulation, Kyushu University, 3-1-1 Maidashi, Higashi-ku, Fukuoka 812-8582, Japan
[2]Laboratory for Proteolytic Neuroscience, RIKEN Brain Science Institute, Saitama, Japan.
[3]Department of Neuropathology, Neurological Institute, Graduate School of Medical Sciences, Kyushu University, 3-1-1 Maidashi, Higashi-ku, Fukuoka 812-8582, Japan
[4]Department of Neuropsychiatry, Graduate School of Medical Sciences, Kyushu University, 3-1-1 Maidashi, Higashi-ku, Fukuoka 812-8582, Japan
[5]Department of Epidemiology and Public Health, Graduate School of Medical Sciences, Kyushu University, 3-1-1 Maidashi, Higashi-ku, Fukuoka 812-8582, Japan
[6]Hisayama Research Institute for Lifestyle Diseases, Hisayama, Fukuoka Japan
[7]Department of Neurobiology and Behavior, University of California, Irvine, CA 92697, USA

[†]Present address: Department of Neurology, University of California, San Francisco, San Francisco, CA 94158, USA.
[§]Present address: Department of Neurosurgery, Japan Community Health care Organization Kyushu Hospital, Kitakyushu, 806-8501, Japan

Correspondence and requests for materials should be addressed to Y.N.
(email: yusaku@bioreg.kyushu-u.c.jp, phone: +81-92-642-6800, FAX: +81-92-642-6791))

This file contains:
Supplementary Figures S1 –S10
Supplementary References

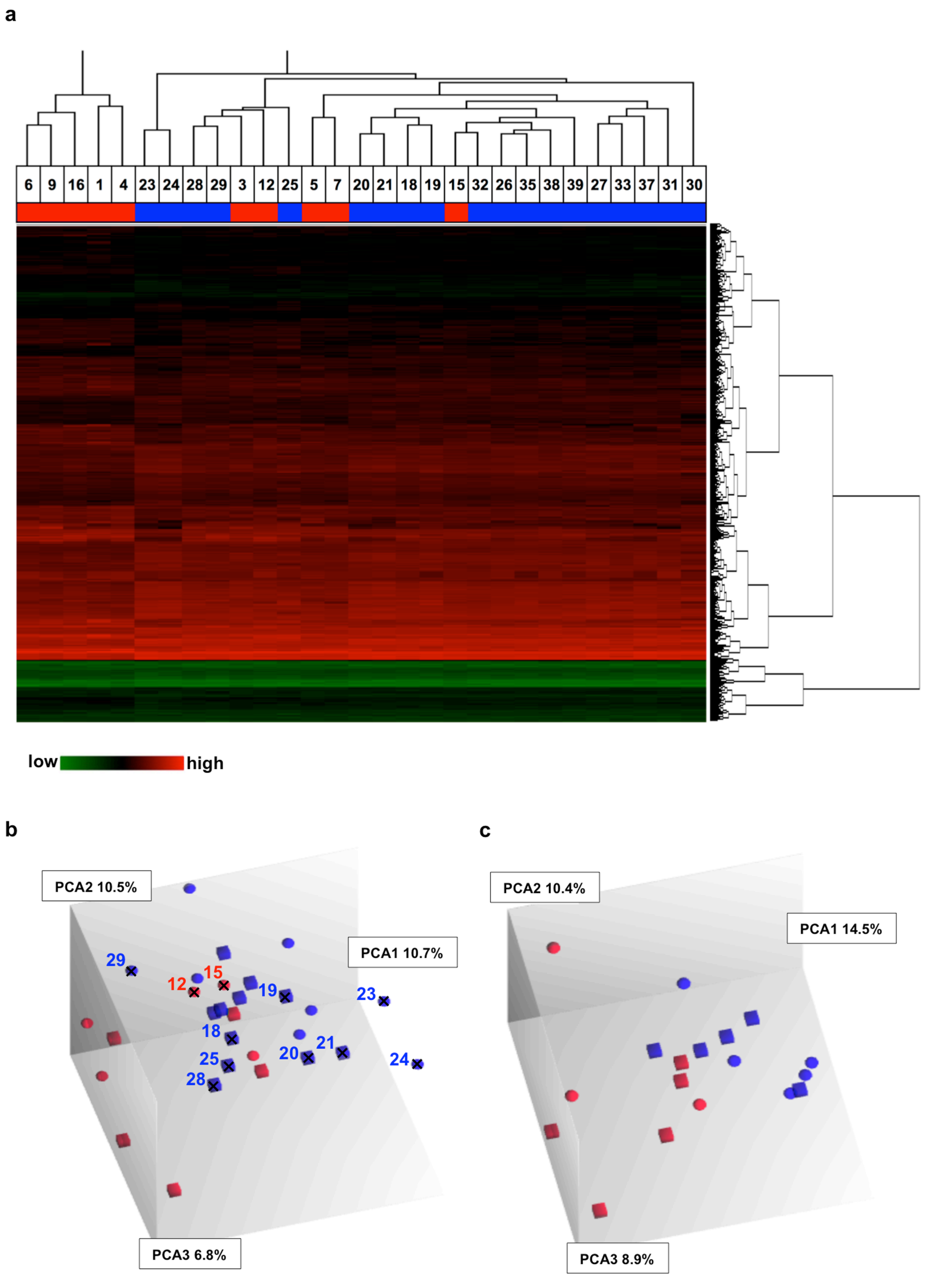

Supplementary Figure S1. Gene expression profiles in human temporal cortices of AD patients. (**a**) Hierarchical clustering. Lists of transcript clusters, which exhibit significant alteration between temporal cortices from AD patients and non-AD subjects obtained from microarray data (ANOVA: $P < 0.05$, fold change ≥ 1.2 or ≤ -1.2), were subjected to hierarchical clustering analysis. Red columns indicate data from AD patients (n = 10), blue columns represent data from non-AD subjects (n = 19). Levels of gene expression are shown in green (low) to red (high). The number at the top of each column indicates the subject ID used for the Hisayama study[9]. **(b, c)** Principal component analysis (PCA). CHP files created from CEL files were subjected to PCA using Affymetrix Expression Console software; graphs with the top three variables (PCA1, PCA2, PCA3) are shown. AD, red; non-AD, blue; cubes, males; spheres, females; number indicates the ID of overlapping subjects shown in (**a**). Overlapping subjects were excluded and the remaining subjects show good segregation (**c**, for PCA; see Fig. 1**a**, for hierarchical clustering) and were used for further analysis.

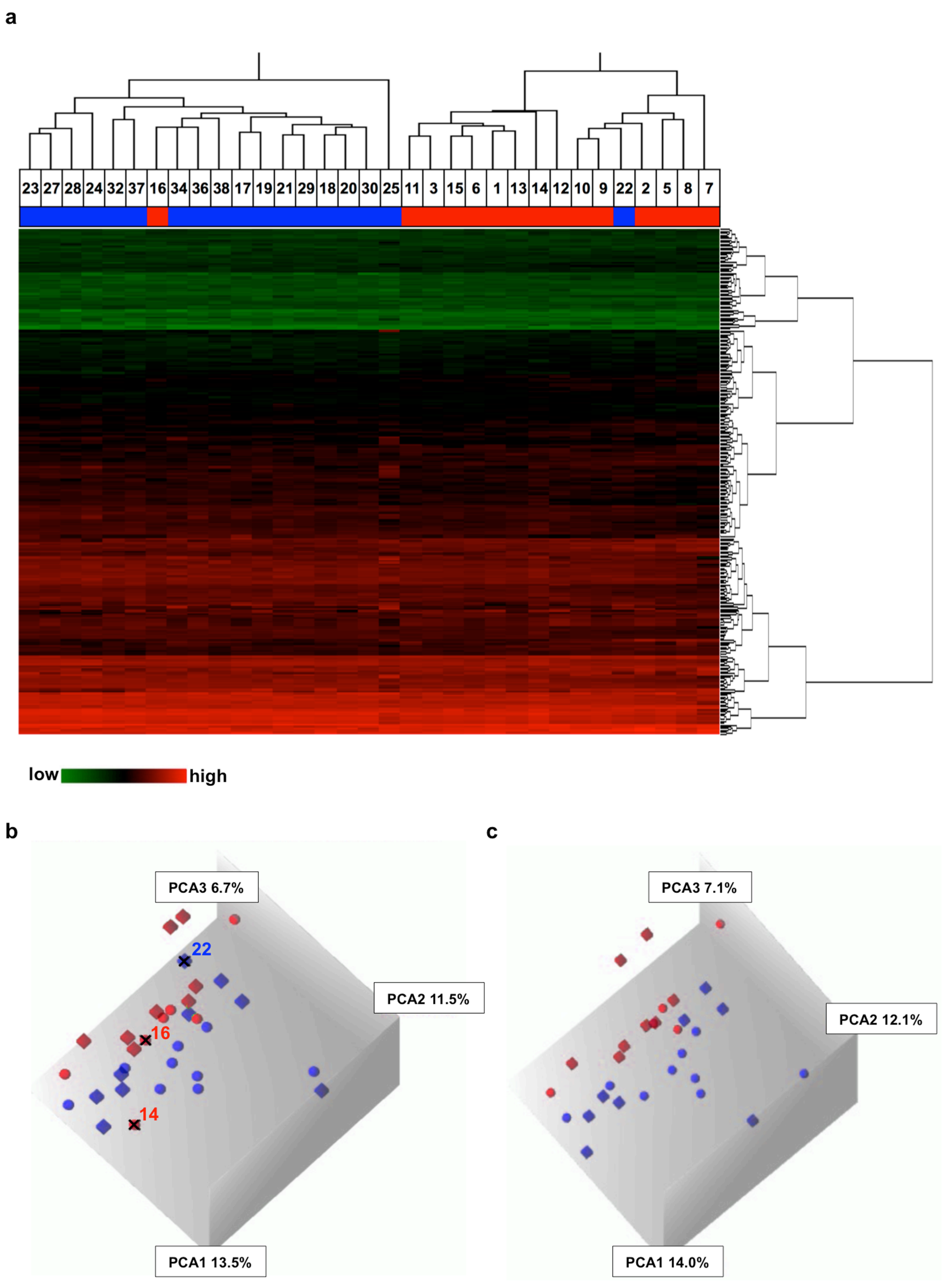

Supplementary Figure S2. Gene expression profiles in human frontal cortices of AD patients. (a) Hierarchical clustering. Lists of transcript clusters, which exhibit significant alteration between frontal cortices from AD patients and non-AD subjects obtained from microarray data (ANOVA: $P < 0.05$, fold change ≥ 1.2 or ≤ -1.2), were subjected to hierarchical clustering analysis. Red columns indicate data from AD patients (n = 15), blue columns represent data from non-AD subjects (n = 18). Levels of gene expression are shown in green (low) to red (high). Number at the top of each column indicates the subject ID used for the Hisayama study[9]. **(b, c)** Principal component analysis (PCA). CHP files created from CEL files were subjected to PCA using Affymetrix Expression Console software; graphs with the top three variables (PCA1, PCA2, PCA3) are shown. AD, red; non-AD, blue; cubes, male; spheres, females; number indicates the ID of overlapping subjects shown in (**a**). Overlapping subjects were excluded and remaining subjects show good segregation (**c**, for PCA; see Fig. 1**b**, for hierarchical clustering) and were used for further analysis.

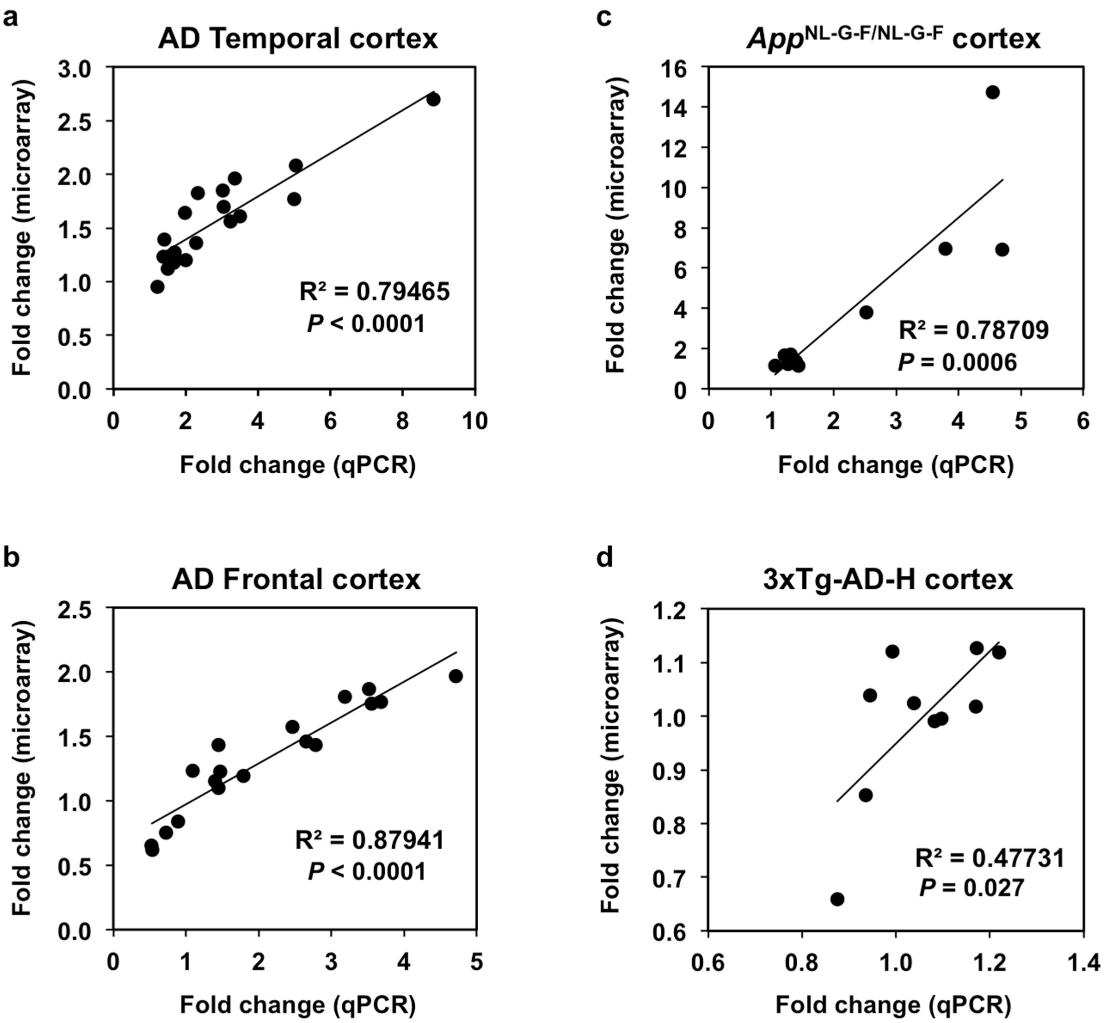

Supplementary Figure S3. Correlation of microarray data with qRT-PCR data. A total of 10 genes, the expression of which was commonly altered in both human temporal cortex (AD vs non-AD) and mouse cortex (*App*$^{NL-G-F/NL-G-F}$ vs WT) as shown in Fig. 4a and c, were selected for validation analysis of human and mouse samples: *C4A*, *C4B*, *CD74*, *CTSS*, *GFAP*, *PHYHD1*, *S100B*, *TF, TGFBR2* and *VIM*. For human samples, microarray data was individually obtained, and *C4A* and *C4B* fold-change values in qRT-PCR were combined to match microarray data. Analysis of variance: (**a**) human temporal cortex (R^2 = 0.79465, *P*-value < 0.0001), (**b**) human frontal cortex (R^2 = 0.87941, *P*-value < 0.0001), (**c**) *App*$^{NL-G-F/NL-G-F}$ mouse cortex (R^2 = 0.78709, *P*-value = 0.0006) and (**d**) 3xTg-AD-H mouse cortex (R^2 = 0.47731, *P*-value = 0.0270).

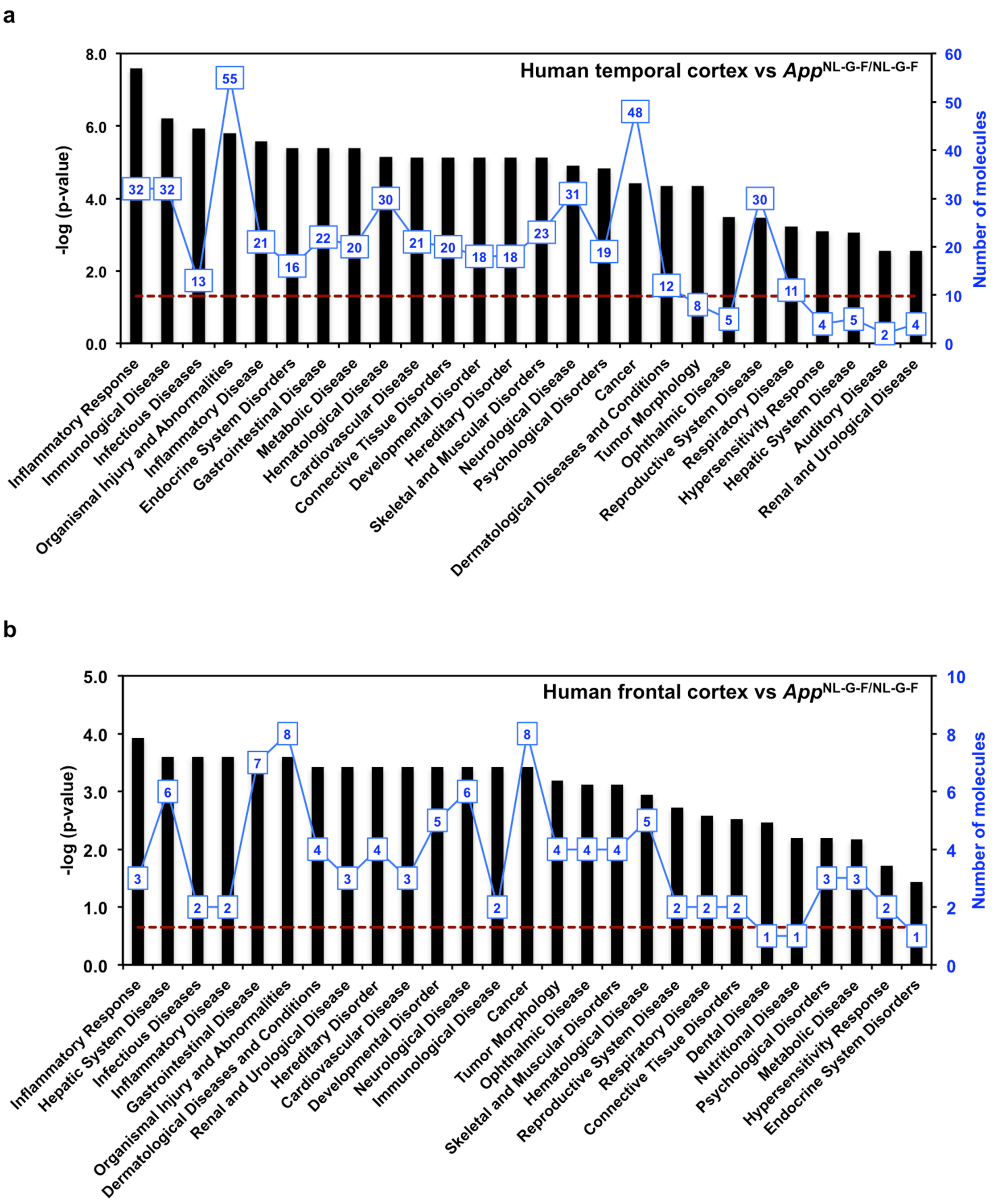

Supplementary Figure S4. Biological functions of commonly altered genes in cortices of human AD subjects and ***App***$^{\mathbf{NL\text{-}G\text{-}F/NL\text{-}G\text{-}F}}$ **mouse models.** Lists of commonly altered genes between *App*$^{NL\text{-}G\text{-}F/NL\text{-}G\text{-}F}$ and human cortices (frontal and temporal), shown in Supplementary Table S8, were input in the IPA software and Core Analysis was performed. Graphs show the biological functions significantly modulated by shared genes between human temporal cortex and *App*$^{NL\text{-}G\text{-}F/NL\text{-}G\text{-}F}$ mouse cortex **(a)**, and between human frontal cortex and *App*$^{NL\text{-}G\text{-}F/NL\text{-}G\text{-}F}$ mouse cortex **(b)**. Black bars indicate the *P*-value (–log(*P*-value)); blue lines indicate the number of molecules categorized in each biological function. Red dashed line indicates the threshold for *P*-value (–log(*P*-value) = 1.3).

a

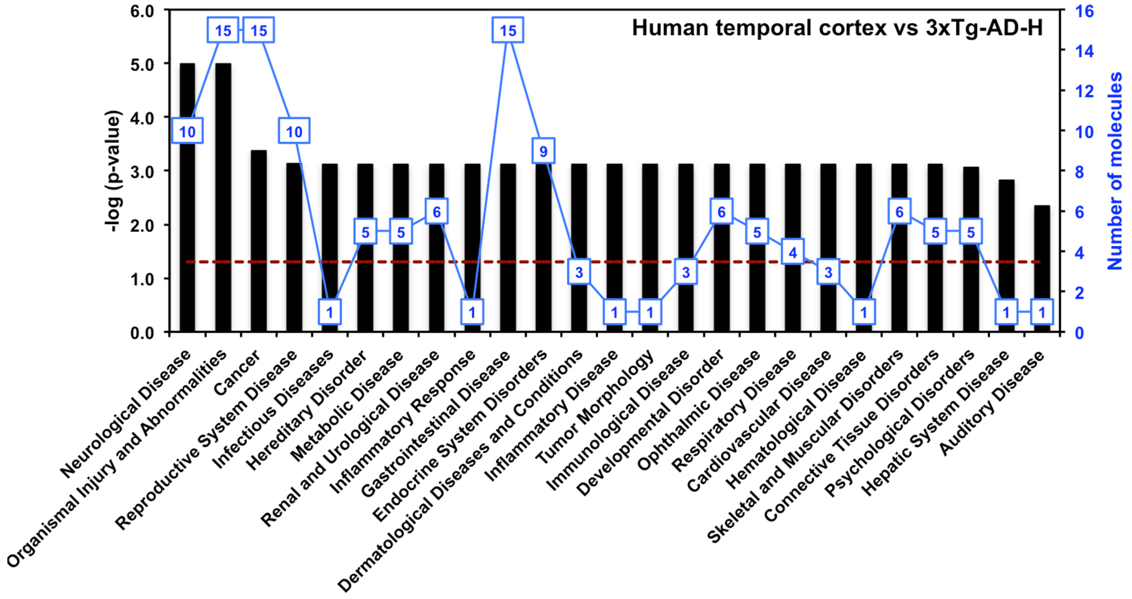

b

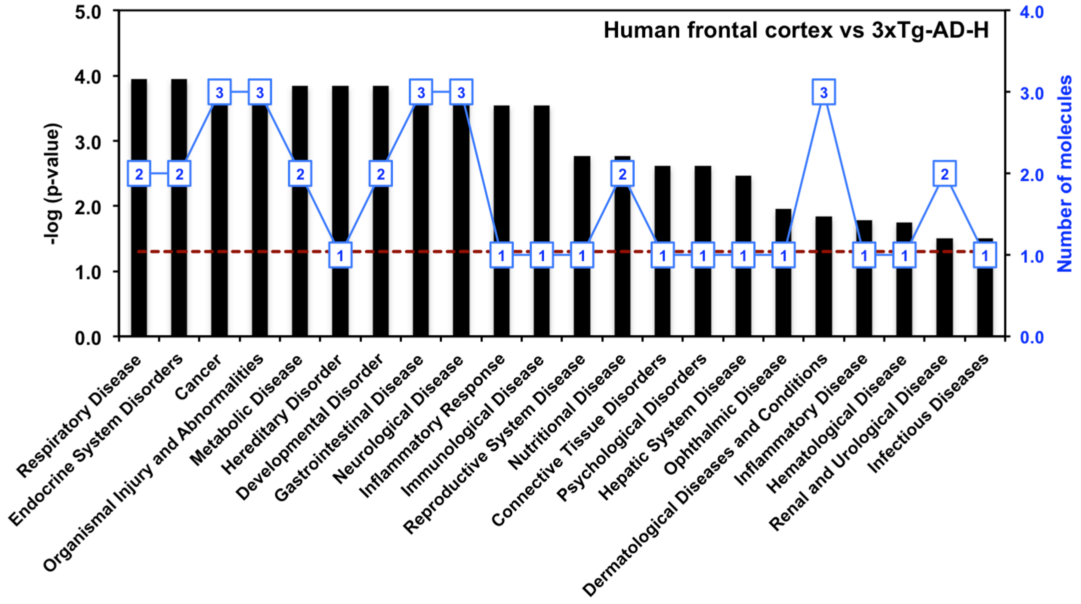

Supplementary Figure S5. Biological functions of commonly altered genes in cortices of human AD subjects and 3xTg-AD-H mouse models. Lists of commonly altered genes between 3xTg-AD-H and human cortices (frontal and temporal), shown in Supplementary Table S9, were input in the IPA software and Core Analysis was performed. Graphs show the biological functions significantly modulated by shared genes between human temporal cortex and 3xTg-AD-H mouse cortex **(a)**, and between human frontal cortex and 3xTg-AD-H mouse cortex **(b)**. Black bars indicate the *P*-value (–log(*P*-value)); blue lines indicate the number of molecules categorised in each biological function. Red dashed line indicates the threshold for p-value (–log(*P*-value) = 1.3).

C4b

Source	DF	SS	F Ratio	Prob > F
Sex	1	1.798	96.421	<.0001
Age	2	55.524	1488.584	<.0001
Genotype	1	70.755	3793.835	<.0001
Sex*Age	2	0.482	12.926	<.0001
Age*Genotype	2	44.274	1186.961	<.0001
Genotype*Sex	1	0.703	37.717	<.0001
Genotype*Sex*Age	2	0.311	8.343	0.0005

Cd74

Source	DF	SS	F Ratio	Prob > F
Sex	1	0.668	6.453	0.0127
Age	2	38.024	183.726	<.0001
Genotype	1	55.708	538.344	<.0001
Sex*Age	2	1.809	8.740	0.0003
Age*Genotype	2	36.936	178.466	<.0001
Genotype*Sex	1	0.778	7.514	0.0073
Genotype*Sex*Age	2	1.717	8.297	0.0005

Ctss

Source	DF	SS	F Ratio	Prob > F
Sex	1	0.056	15.103	0.0002
Age	2	4.307	579.933	<.0001
Genotype	1	14.388	3874.850	<.0001
Sex*Age	2	0.017	2.289	0.1069
Age*Genotype	2	3.533	475.787	<.0001
Genotype*Sex	1	0.014	3.890	0.0514
Genotype*Sex*Age	2	0.013	1.730	0.1828

Gfap

Source	DF	SS	F Ratio	Prob > F
Sex	1	1.113	163.765	<.0001
Age	2	10.499	772.397	<.0001
Genotype	1	26.808	3944.353	<.0001
Sex*Age	2	0.291	21.426	<.0001
Age*Genotype	2	10.361	762.228	<.0001
Genotype*Sex	1	0.723	106.412	<.0001
Genotype*Sex*Age	2	0.334	24.596	<.0001

Nfe2l2

Source	DF	SS	F Ratio	Prob > F
Sex	1	0.001	0.148	0.7010
Age	2	3.214	160.911	<.0001
Genotype	1	5.993	600.101	<.0001
Sex*Age	2	0.042	2.111	0.1267
Age*Genotype	2	1.323	66.232	<.0001
Genotype*Sex	1	0.017	1.665	0.2000
Genotype*Sex*Age	2	0.068	3.398	0.0375

Phyhd1

Source	DF	SS	F Ratio	Prob > F
Sex	1	0.004	0.457	0.5008
Age	2	1.016	62.736	<.0001
Genotype	1	3.187	393.498	<.0001
Sex*Age	2	0.100	6.172	0.003
Age*Genotype	2	1.066	65.821	<.0001
Genotype*Sex	1	0.080	9.936	0.0022
Genotype*Sex*Age	2	0.004	0.240	0.7869

S100b

Source	DF	SS	F Ratio	Prob > F
Sex	1	0.169	60.786	<.0001
Age	2	0.850	152.893	<.0001
Genotype	1	0.947	340.671	<.0001
Sex*Age	2	0.245	44.127	<.0001
Age*Genotype	2	0.171	30.695	<.0001
Genotype*Sex	1	0.001	0.406	0.5256
Genotype*Sex*Age	2	0.011	1.896	0.1557

Tf

Source	DF	SS	F Ratio	Prob > F
Sex	1	0.477	92.465	<.0001
Age	2	3.563	345.688	<.0001
Genotype	1	2.598	504.182	<.0001
Sex*Age	2	0.786	76.269	<.0001
Age*Genotype	2	0.790	76.680	<.0001
Genotype*Sex	1	0.133	25.780	<.0001
Genotype*Sex*Age	2	0.144	13.980	<.0001

Tgfbr2

Source	DF	SS	F Ratio	Prob > F
Sex	1	0.741	77.476	<.0001
Age	2	1.102	57.672	<.0001
Genotype	1	9.514	995.386	<.0001
Genotype*Age	2	1.308	68.411	<.0001
Age*Sex	2	0.299	15.664	<.0001
Genotype*Sex	1	0.366	38.290	<.0001
Genotype*Sex*Age	2	0.186	9.738	0.0001

Vim

Source	DF	SS	F Ratio	Prob > F
Sex	1	2.143	106.663	<.0001
Age	2	0.107	2.674	0.0741
Genotype	1	0.523	26.004	<.0001
Sex*Age	2	0.399	9.922	0.0001
Age*Genotype	2	0.897	22.324	<.0001
Genotype*Sex	1	0.554	27.578	<.0001
Genotype*Sex*Age	2	0.165	4.109	0.0194

Supplementary Figure S6. Results of effect test of 3-way ANOVA for data shown in Figure 5. The results of effect tests of 3-way ANOVA performed for qRT-PCR data of the 10 genes presented in Fig. 5 are shown. Significant *P*-value for Prob > F are indicated in red ($\alpha = 0.05$). DF: Degree of Freedom. SS: Sum of Squares. Sex: male, female. Age: 5, 7 and 12 months of age. Genotype: $App^{NL\text{-}G\text{-}F/NL\text{-}G\text{-}F}$ and wild-type.

Level – *C4b*

Level	
Female *App*$^{NL-G-F/NL-G-F}$, 12	A
Male *App*$^{NL-G-F/NL-G-F}$, 12	B
Female *App*$^{NL-G-F/NL-G-F}$, 7	C
Male *App*$^{NL-G-F/NL-G-F}$, 7	D
Female *App*$^{NL-G-F/NL-G-F}$, 5	E
Male *App*$^{NL-G-F/NL-G-F}$, 5	E F
Female WT, 12	E F G
Male WT, 12	F G H
Female WT, 5	F G H I
Female WT, 7	G H I
Male WT, 5	H I
Male WT, 7	I

Level – *Cd74*

Level	
Female *App*$^{NL-G-F/NL-G-F}$, 12	A
Male *App*$^{NL-G-F/NL-G-F}$, 12	A
Male *App*$^{NL-G-F/NL-G-F}$, 7	B
Female *App*$^{NL-G-F/NL-G-F}$, 7	C
Male *App*$^{NL-G-F/NL-G-F}$, 5	D
Female *App*$^{NL-G-F/NL-G-F}$, 5	D
Female WT, 12	D
Male WT, 12	D
Female WT, 5	D
Female WT, 7	D
Male WT, 5	D
Male WT, 7	D

Level – *Ctss*

Level	
Female *App*$^{NL-G-F/NL-G-F}$, 12	A
Male *App*$^{NL-G-F/NL-G-F}$, 12	B
Female *App*$^{NL-G-F/NL-G-F}$, 7	C
Male *App*$^{NL-G-F/NL-G-F}$, 7	D
Female *App*$^{NL-G-F/NL-G-F}$, 5	E
Male *App*$^{NL-G-F/NL-G-F}$, 5	E
Female WT, 12	F
Male WT, 12	F G
Female WT, 7	F G
Female WT, 5	F G
Male WT, 7	G
Male WT, 5	G

Level – *Gfap*

Level	
Female *App*$^{NL-G-F/NL-G-F}$, 12	A
Male *App*$^{NL-G-F/NL-G-F}$, 12	B
Female *App*$^{NL-G-F/NL-G-F}$, 7	C
Male *App*$^{NL-G-F/NL-G-F}$, 7	D
Female *App*$^{NL-G-F/NL-G-F}$, 5	E
Male *App*$^{NL-G-F/NL-G-F}$, 5	F
Female WT, 5	G
Female WT, 12	G
Male WT, 12	G
Female WT, 7	G
Male WT, 5	G
Male WT, 7	G

Level – *Nfe2l2*

Level	
Female *App*$^{NL-G-F/NL-G-F}$, 12	A
Male *App*$^{NL-G-F/NL-G-F}$, 12	A
Male *App*$^{NL-G-F/NL-G-F}$, 7	B
Female *App*$^{NL-G-F/NL-G-F}$, 7	B
Male *App*$^{NL-G-F/NL-G-F}$, 5	B C
Female *App*$^{NL-G-F/NL-G-F}$, 5	B C
Male WT, 12	C D
Female WT, 12	C D E
Male WT, 7	D E
Female WT, 5	D E
Female WT, 7	E
Male WT, 5	E

Level – *Phyhd1*

Level	
Male *App*$^{NL-G-F/NL-G-F}$, 12	A
Female *App*$^{NL-G-F/NL-G-F}$, 12	A
Male *App*$^{NL-G-F/NL-G-F}$, 7	B
Female *App*$^{NL-G-F/NL-G-F}$, 7	C
Female *App*$^{NL-G-F/NL-G-F}$, 5	D
Male *App*$^{NL-G-F/NL-G-F}$, 5	D
Female WT, 5	D
Female WT, 12	D
Male WT, 12	E
Male WT, 5	E
Male WT, 7	E
Female WT, 7	E

Level – *S100b*

Level	
Female *App*$^{NL-G-F/NL-G-F}$, 12	A
Male *App*$^{NL-G-F/NL-G-F}$, 12	B
Male *App*$^{NL-G-F/NL-G-F}$, 7	C
Female WT, 12	C
Female *App*$^{NL-G-F/NL-G-F}$, 7	C
Female *App*$^{NL-G-F/NL-G-F}$, 5	D
Male *App*$^{NL-G-F/NL-G-F}$, 5	E
Male WT, 7	E F
Female WT, 5	E F
Male WT, 12	E F
Female WT, 7	F
Male WT, 5	G

Level – *Tf*

Level	
Female *App*$^{NL-G-F/NL-G-F}$, 12	A
Male *App*$^{NL-G-F/NL-G-F}$, 12	B
Male *App*$^{NL-G-F/NL-G-F}$, 7	C
Female WT, 12	C
Female *App*$^{NL-G-F/NL-G-F}$, 7	C
Female *App*$^{NL-G-F/NL-G-F}$, 5	D
Male WT, 12	D E
Male *App*$^{NL-G-F/NL-G-F}$, 5	D E F
Male WT, 7	D E F
Female WT, 5	E F
Female WT, 7	F
Male WT, 5	F

Level – *Tgfbr2*

Level	
Female *App*$^{NL-G-F/NL-G-F}$, 12	A
Female *App*$^{NL-G-F/NL-G-F}$, 7	B
Male *App*$^{NL-G-F/NL-G-F}$, 7	B C
Male *App*$^{NL-G-F/NL-G-F}$, 12	C D
Female *App*$^{NL-G-F/NL-G-F}$, 5	D
Male *App*$^{NL-G-F/NL-G-F}$, 5	E
Female WT, 7	F
Female WT, 5	F
Female WT, 12	F
Male WT, 7	F
Male WT, 5	F
Male WT, 12	F

Level – *Vim*

Level	
Female *App*$^{NL-G-F/NL-G-F}$, 7	A
Female *App*$^{NL-G-F/NL-G-F}$, 5	B
Female *App*$^{NL-G-F/NL-G-F}$, 12	B
Female WT, 5	B C
Male *App*$^{NL-G-F/NL-G-F}$, 12	B C D
Female WT, 7	B C D E
Male WT, 5	C D E F
Male WT, 7	D E F G
Female WT, 12	D E F G
Male *App*$^{NL-G-F/NL-G-F}$, 7	E F G
Male WT, 12	F G
Male *App*$^{NL-G-F/NL-G-F}$, 5	G

Supplementary Figure S7. Results of post-hoc Turkey HSD test for data shown in Figure 5. The results of post-hoc Turkey HSD tests performed for qRT-PCR data of the 10 genes presented in Fig. 5 are shown. Levels not connected by the same letter are significantly different (*P*-value < 0.05).

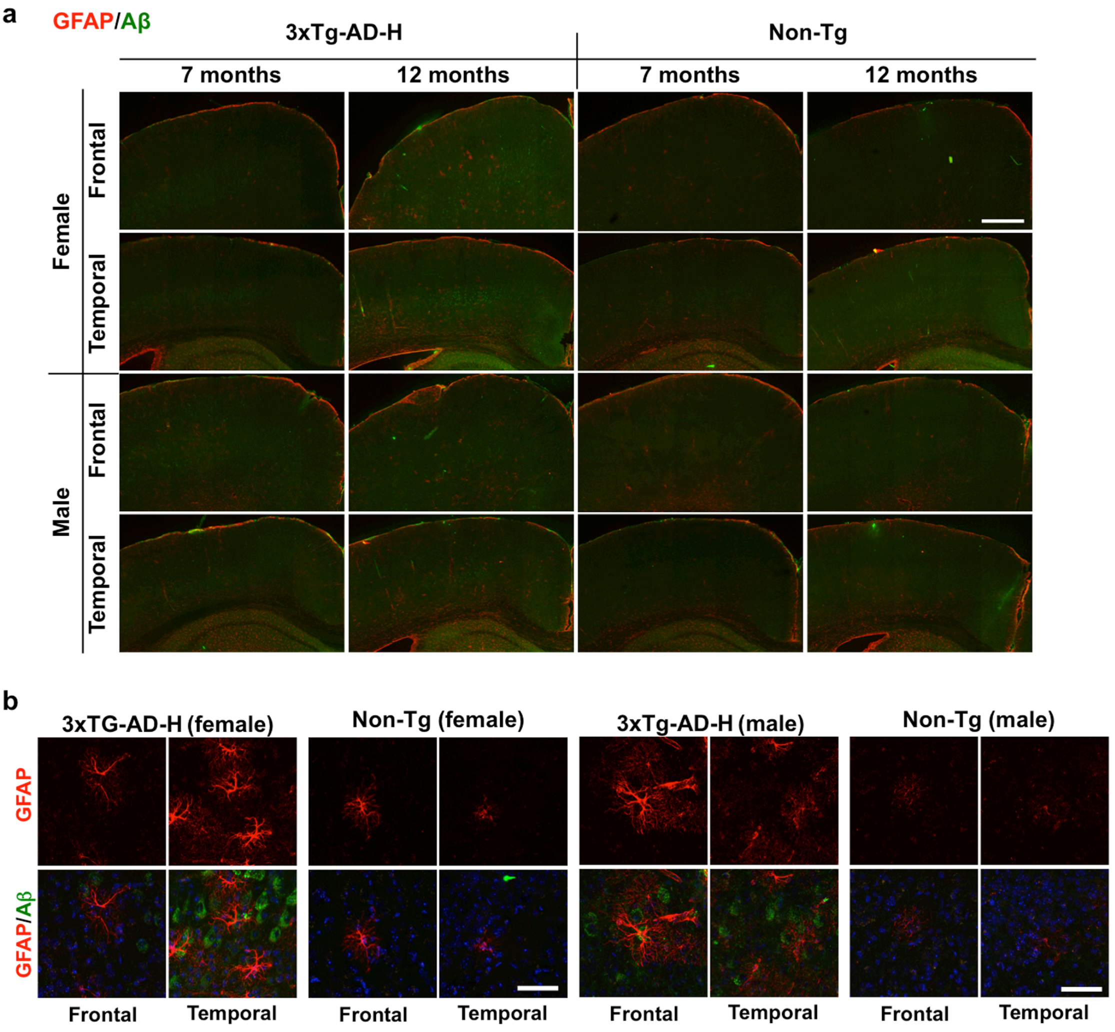

Supplementary Figure S8. Double-immunofluorescence microscopy for Aβ and GFAP in the 3xTg-AD-H mouse model. Coronal sections containing frontal (Bregma: –1.255 to –1.455) and temporal (Bregma: +1.845 to +2.045) cortices prepared from 7- and 12-month-old, male and female 3xTg-AD-H and non-Tg mice, were subjected to double-immunofluorescence microscopy using mouse anti-human Aβ (green) and rabbit anti-GFAP antibodies (red). (**a**) Multiple z-stack images of 15 fields were tiled and stacked together using ZEN imaging software. Scale bar = 500 µm. (**b**) Magnified images from 12-month-old samples. Scale bar = 50 µm.

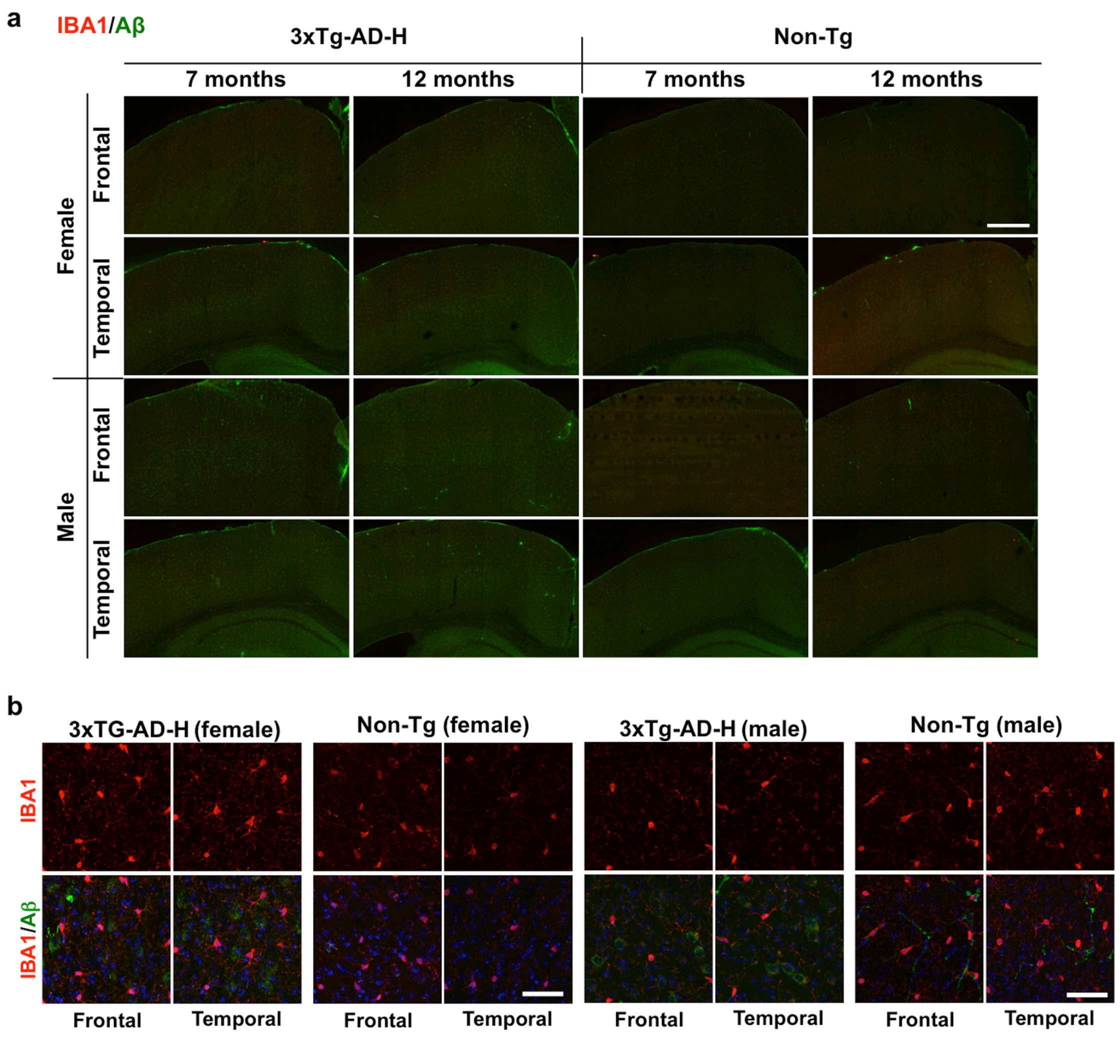

Supplementary Figure S9. Double-immunofluorescence microscopy for Aβ and IBA1 in the 3xTg-AD-H mouse model. Coronal sections containing frontal (Bregma: –1.255 to –1.455) and temporal (Bregma: +1.845 to +2.045) cortices prepared from 7- and 12-month-old, male and female 3xTg-AD-H and non-Tg mice, were subjected to double-immunofluorescence microscopy using mouse anti-human Aβ (green) and rabbit anti-IBA1 antibodies (red). (**a**) Multiple z-stack images of 15 fields were tiled and stacked together using ZEN imaging software. Scale bar = 500 µm. (**b**) Magnified images from 12-month-old samples. Scale bar = 50 µm.

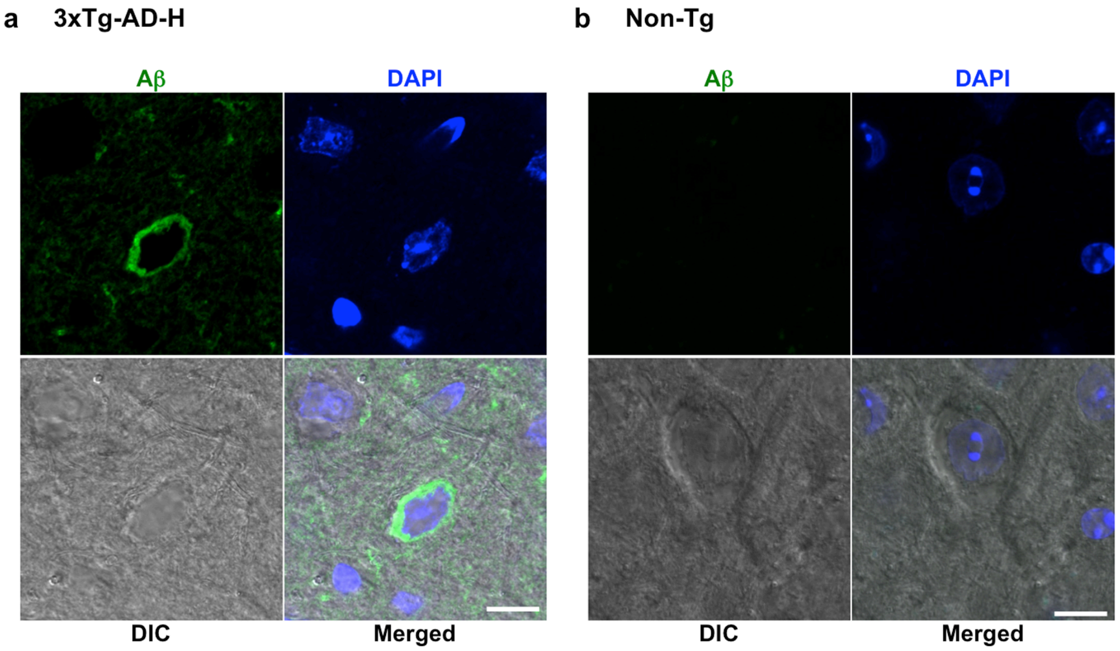

Supplementary Figure S10. Laser scanning immunofluorescence microscopy with differential interference contrast for Aβ in the 3xTg-AD-H mouse model. Coronal sections containing temporal cortices prepared from 12-month-old male 3xTg-AD-H (a) and non-Tg (b) mice were subjected to laser scanning immunofluorescence microscopy with differential interference contrast (DIC) using mouse anti-human Aβ (green) antibody. Nuclei were counterstained with DAPI (blue) Scale bar = 10 μm.

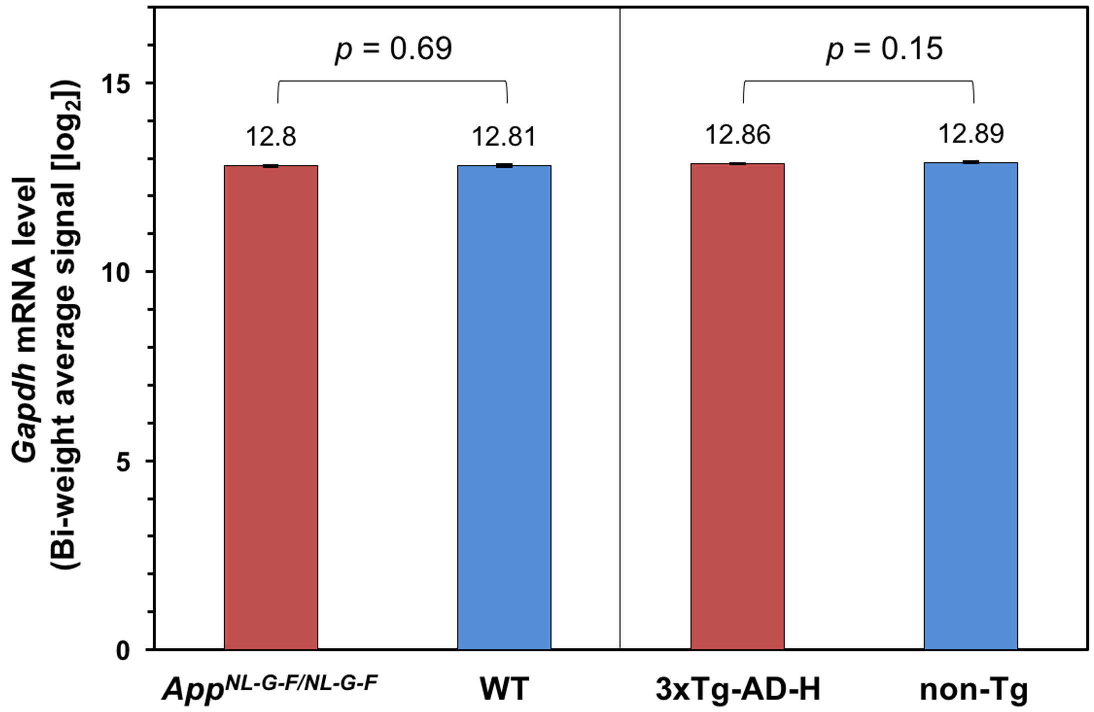

Supplementary Figure S11. *Gapdh* mRNA levels do not change between *App*$^{NL\text{-}G\text{-}F/NL\text{-}G\text{-}F}$ and WT, or 3xTg-AD-H and non-Tg cortices. Expression levels of *Gapdh* mRNA in *App*$^{NL\text{-}G\text{-}F/NL\text{-}G\text{-}F}$, WT, 3xTg-AD-H and non-Tg cortex obtained from microarray data are shown as the Bi-weight average signal [$\log_2$]. *P*-values are from one-way between subject ANOVA (TAC software).

Supplementary references

1. Zhu JB, Tan CC, Tan L, Yu JT. State of Play in Alzheimer's Disease Genetics. *J Alzheimers Dis* 58, 631-659 (2017).
2. Zhang B*, et al.* Integrated systems approach identifies genetic nodes and networks in late-onset Alzheimer's disease. *Cell* 153, 707-720 (2013).
3. Matarin M*, et al.* A genome-wide gene-expression analysis and database in transgenic mice during development of amyloid or tau pathology. *Cell Rep* 10, 633-644 (2015).
4. Lambert JC*, et al.* Meta-analysis of 74,046 individuals identifies 11 new susceptibility loci for Alzheimer's disease. *Nat Genet* 45, 1452-1458 (2013).
5. Sims R*, et al.* Rare coding variants in PLCG2, ABI3, and TREM2 implicate microglial-mediated innate immunity in Alzheimer's disease. *Nat Genet*, doi: 10.1038/ng.3916. [Epub ahead of print] (2017).
6. Naj AC, Schellenberg GD, Alzheimer's Disease Genetics C. Genomic variants, genes, and pathways of Alzheimer's disease: An overview. *Am J Med Genet B Neuropsychiatr Genet* 174, 5-26 (2017).
7. Efthymiou AG, Goate AM. Late onset Alzheimer's disease genetics implicates microglial pathways in disease risk. *Mol Neurodegener* 12, 43 (2017).
8. Walter S*, et al.* A genome-wide association study of aging. *Neurobiol Aging* 32, 2109 e2115-2128 (2011).
9. Hokama M, et al. Altered Expression of Diabetes-Related Genes in Alzheimer's Disease Brains: The Hisayama Study. Cereb Cortex 24, 2476-2488 (2014).
